# Three-tiered authentication of herbal traditional Chinese medicine ingredients used in women’s health provides progressive qualitative and quantitative insight

**DOI:** 10.3389/fphar.2024.1353434

**Published:** 2024-02-05

**Authors:** Felicitas Mück, Francesca Scotti, Quentin Mauvisseau, Birgitte Lisbeth Graae Thorbek, Helle Wangensteen, Hugo J. de Boer

**Affiliations:** ^1^ Section for Pharmaceutical Chemistry, Department of Pharmacy, University of Oslo, Oslo, Norway; ^2^ Department of Pharmaceutical and Biological Chemistry, School of Pharmacy, University College London, London, United Kingdom; ^3^ Natural History Museum, University of Oslo, Oslo, Norway

**Keywords:** chemical fingerprinting, DNA barcoding, endometriosis, pharmacovigilance, Traditional Chinese Medicine, women’s healthcare

## Abstract

Traditional Chinese Medicine (TCM) herbal products are increasingly used in Europe, but prevalent authentication methods have significant gaps in detection. In this study, three authentication methods were tested in a tiered approach to improve accuracy on a collection of 51 TCM plant ingredients obtained on the European market. We show the relative performance of conventional barcoding, metabarcoding and standardized chromatographic profiling for TCM ingredients used in one of the most diagnosed disease patterns in women, endometriosis. DNA barcoding using marker ITS2 and chromatographic profiling are methods of choice reported by regulatory authorities and relevant national pharmacopeias. HPTLC was shown to be a valuable authentication tool, combined with metabarcoding, which gives an increased resolution on species diversity, despite dealing with highly processed herbal ingredients. Conventional DNA barcoding as a recommended method was shown to be an insufficient tool for authentication of these samples, while DNA metabarcoding yields an insight into biological contaminants. We conclude that a tiered identification strategy can provide progressive qualitative and quantitative insight in an integrative approach for quality control of processed herbal ingredients.

## Introduction

Traditional Chinese Medicine (TCM) is one of the most established traditional medical systems and an increasingly popular health resource throughout the world (Wang et al., 2019; World Health Organization, 2022). TCMs have been widely used in the treatment of acute and chronic diseases over thousands of years (Li et al., 2019). TCM is categorized as a Complementary and Alternative Medicine (CAM) (World Health Organization, 2013), which includes amongst other sub-categories Chinese Herbal Medicines (CHMs). Gynecology is one of the main branches in TCM and harbors a long history in the treatment and management of gynecological disorders (Zell et al., 2000; Maciocia, 2011; Zhang et al., 2011; Chen et al., 2020). A common health condition for women is severe chronic pain in the pelvic area, and associated disease patterns often fall under the clinical diagnosis of dysmenorrhea, or endometriosis associated with complex symptoms (Reid et al., 2019; Nie et al., 2020; Taylor et al., 2021). A recent large-scale randomized and placebo-controlled trial confirmed the efficacy and safety of specific CHMs as a treatment for endometriosis-associated pain and related symptoms (Lin et al., 2022).

There are unique considerations to be made for the cultivation and processing of herbal ingredients for medicine, as a characteristic part of the ethnopharmacology of TCM (Guo et al., 2015). In the foreground are *Daodi* cultivation, which is linked with high quality material from specific geographical regions, and *Paozhi* processing where raw plant material is processed into decoction pieces which are treated with excipients that fundamentally alter their metabolic profile resulting in changed bioactivity levels, such as enhanced efficacy, modified medicinal properties and reduced toxicity (Shaw, 2010; Guo et al., 2015; Engelhardt et al., 2018; Wu et al., 2018; Wang et al., 2019). Chinese medicinal processing, as an integral part of TCM, is a pharmaceutical technique in order to meet therapeutic, dispensing and preparation requirements (Guo et al., 2015). Depending on wanted individualized medicinal properties from specific plants, a variety of techniques are used in TCM processing. First simple preparation, like cleaning and cutting of herbs, and second exhaustive processing, like stir-frying, stir-frying with liquids or solid adjuvants, steaming with salt water, medicinal juices, vinegar or wine, boiling and calcining with mineral salts, alum or fresh ginger (Guo et al., 2015). Generally, the choice for which processing method is used as a standard for which ingredient remains controversial, and methods for plant specific Chinese medicinal processing are evolving. Nonetheless, decoction pieces produced with ingredient specific processing techniques are indiscriminately prescribed in proprietary TCMs and in prescription handed out by TCM practitioners (Guo et al., 2015). Interestingly, TCM ingredients are described with herbal drug names defining a plant’s genus and the part of the plant used. However, the ingredient name is seldom coherent with scientific taxonomy and ingredient names often refer to more than one plant species (Mück et al., 2023). The Chinese Pharmacopoeia states which species are accepted under the ingredient name with scientific taxonomy (Chinese Pharmacopoeia Committee, 2020).

The quality of TCM materials, their safety and therapeutic efficacy are of critical importance. Quality risks in CHM are related to authenticity issues through misidentification, or mislabeling of herbal ingredients, adulterations, and substitutions, or endogenous and exogenous substances, caused by improper processing of herbs, or heavy metal, pesticides and microbial contaminations (He et al., 2015; Wu et al., 2018; Chen et al., 2023). As examples, a study investigating 400 seeds for TCM manufacturing detected that 7.5% of the seeds where incorrectly labeled (Xiong et al., 2018), and in another study by Xu et al. (2019), 166 adulterants were detected from various TCMs. Good manufacturing practice (GMP), good agricultural and collection practices (GACP), good plant authentication and identification practice (GPAIP), and good laboratory practices (GLP) guidelines in TCM are regarded as important tools to meet good quality requirements (Govindaraghavan, 2008; Zhang et al., 2012; Govindaraghavan and Sucher, 2015; He et al., 2015). There are challenges in the “internationalization of TCM” including *inter alia* difficulties in quality control, legislative barriers in marketing TCMs and unclear basis of therapeutic mechanisms (Lin et al., 2018). Current quality control of TCMs including processed herbal preparations and products is in great dispute because, unlike chemical drugs, we lack clear quality standards for TCMs, and analytical methods applying qualitative markers are not integral enough to assess their complex nature. Despite established monographs and standards for quality control, processed products, established preparations in pharmacopeias and directives for identification, tests, assays, and definitions, etc., the information for clear differentiation of closely related, or similar species is not enough (Chan et al., 2009; Bauer and Franz, 2010; Li et al., 2019). The factors for high quality of herbal materials are furthermore very complex leading to technical challenges for regulatory authorities when formulating guidelines, resulting in different regulatory requirements across regions and countries (He et al., 2015; Wu et al., 2018). Besides, juristic and marketing differences among countries contribute to poor regulations and subsequently difficulties on quality assurance of herbal products (Ichim and Booker, 2021).

The development of comprehensive quality standards of CHMs and effective quality control procedures for authenticity testing and standard development of Chinese herbal materials is an ongoing challenge (Li et al., 2019; Leong et al., 2020). To better evaluate the complexity of CHMs, an integrative approach involving effective pharmacological methods, biological and chemical techniques is required. Conventional DNA barcoding systems have been adopted by national pharmacopeias, like the Chinese Pharmacopoeia, British Pharmacopoeia and Japanese Pharmacopoeia (Chen et al., 2017). DNA barcoding techniques, as described in the British Pharmacopoeia (British Pharmacopoeia Commission, 2018), are a valuable screening tool for raw, single species botanical materials. Metabarcoding techniques have been successfully used for ingredient profiling of commercial herbal products, which are composed of varying, processed botanical materials, such as CHMs and provide information about unknown ingredients (Coghlan et al., 2012; Arulandhu et al., 2017; Seethapathy et al., 2019; Anthoons et al., 2021; Zhu et al., 2022; Raclariu-Manolică, 2023a; Raclariu-Manolicăet al., 2023b). Chromatographic authentication gives high resolutions for the detection of target compounds of known ingredients and are basic authentication tools for herbal remedies (Liang et al., 2010; Booker et al., 2016; Heinrich et al., 2022).

The aim of this study was to develop a testing strategy for the authentication of processed herbal ingredients. We investigate different authentication methods looking at the independent CHM ingredients used in two formulae utilized in the context of gynecological health, *Gui Zhi Fu Ling Wan* and *Ge Xia Zhu Yu Tang*. Shared among them these formulae include 13 CHM ingredients corresponding to 17 plant species. We compare the results from authenticating 51 single CHM ingredients, using three different analytical techniques, high-performance thin-layer chromatography (HPTLC), DNA barcoding and DNA metabarcoding.

## Materials and methods

### Sample material

Fifty-one CHM ingredients were collected in 2019 from commercial TCM distributors and online retailers in Europe, listing: Angelica Sinensis Radix (4), Aurantii Fructus (3), Carthami Flos (3), Chuanxiong Rhizoma (4), Cinnamomi Ramulus (3), Corydalis Rhizoma (4), Cyperi Rhizoma (10), Glycyrrhizae Radix (3), Linderae Radix (3), Moutan Cortex (4), Paeoniae Radix Rubra (4), Persicae Semen (4), and Poriae Cocos (2). Samples were sold as single ingredient TCM decoction pieces. The TCM products were imported into Norway for scientific analyses under Norwegian Medicines Agency license ref. no 18/13,493-2. The sample materials were ground and homogenized using an IKA Tube Mill 100 (IKA-Werke GmbH & Co. KG, Staufen, Germany). The sample materials are CHM ingredients of two formulae utilized in the context of gynecological health, *Gui Zhi Fu Ling Wan* (Table 1) and *Ge Xia Zhu Yu Tang* (Table 2). The tables provide an overview of the species, characteristics, and processing techniques of these CHM ingredients.

**TABLE 1:** Properties^a^ of ingredients in Chinese Herbal Medicine formula *Gui Zhi Fu Ling Wan*.

Ingredients	Plant species	Quality indicators for crude materials	Processing method	Known substitutes/adulterants
**Pinyin: Gui Zhi Herbal drug name: Cinnamomi Ramulus**	*Neolitsea cassia* (L.) Kosterm., syn.: *Cinnamomum cassia* (L.) J. Presl	Young twigs without leaves or any withered parts	Twigs are dry-fried or stir fried or baked with honey	*Cinnamomum austro-sinensis* H.T.Chang, *Cinnamomum bejolghota* (Buch.-Ham.) Sweet, *Cinnamomum burmanni* (Nees & T.Nees) Blume, *Cinnamomum heyneanum* Nees, *Cinnamomum japonicum* Siebold, *Cinnamomum subavenium* Miq., *Cinnamomum tamala* (Buch.-Ham.) T.Nees & C.H.Eberm., *Cinnamomum wilsonii* Gamble
**Pinyin: Fu Ling Herbal drug name: Poriae Cocos**	*Poria cocos* (Schw.) Wolf	Hard, solid, white, without inlays of soil, sticks	dry- frying or baking over low heat	Substitution/adulteration not common
**Pinyin: Chi Shao Herbal drug name: Paeoniae Radix Rubra**	*Peonia lactiflora* Pall. and *Paeonia anomala* subsp. Veitchii (Lynch) D.Y. Hong & K.Y. Pan	Long, thick, powdery roots with pale reddish or yellowish cross sections	Dry-frying, wine-frying, or vinegar- frying of slices	*Paeonia obovata* Maxim., *Paeonia obovata* var. willmottiae, *Paeonia anomala* var. intermedia, *Paeonia anomala* L. subsp. anomala *Paeonia mairei* H. Lev, *Sanguisorba officinalis*
**Pinyin: Mu Dan Pi Herbal drug name: Moutan Cortex**	*Paeonia suffruticosa* Andrews	Thick, white, starchy quills with xylem removed and strong aroma	Dry-frying, wine-frying, charred moutan (dry-fried or baked at high heat until blackened)	*Paeonia ostii* T. Hong & J. X. Zhang, *Paeonia delavayi* Franch., *Paeonia decomposita* Hand. -Mazz. subsp. decomposita, *Paeonia anomala* L. subsp. Veitchii (Lynch) D. Y. Hong& K. Y. Pan
**Pinyin: Tao Ren Herbal drug name: Persicae Semen**	*Prunus davidiana* Franch*, Prunus persica* (L.)	Large, flat cut full and closed seeds with white and oily kernels	Stripped peach kernel (clean kernels are boiled and coat rubbed off), ordry-frying, or defatting peach kernel (oils removed from cleaned kernel)	Substitution/adulteration not common

^a^ Information retrieved from Bensky et al. (2004); Scheid et al. (2015); Leon and Yu-Lin (2017).

**TABLE 2:** Properties^a^ of Chinese herbal ingredients in Chinese Herbal Medicine formula *Ge Xia Zhu Yu Tang*.

Ingredients	Plant species	Quality indicators for crude materials	Processing method	Known substitutes/adulterants
**Pinyin: Dang Gui Herbal drug name: Angelicae Sinensis Radix**	*Angelica sinensis* (Oliv.) Diels	Thick, long main roots, yellowish brown, soft outer bark, yellowish white cross section, dense aroma	Dry-frying, wine-frying, earth-frying (with Terra flava usta), charred fried (dry-fried until surface is blackened) of slices	*Levisticum officinale* W.D. J. Koch *Angelica acutiloba* (Siebold& Zucc.) Kitag., *Angelica megaphylla* Diels, *Angelica gigas* Nakai, *Angelica polymorpha* Maxim., *Ligusticum glaucescens* Franch. and Hansenia forbesii (H.Boissieu) Pimenov & Kljuykov
**Pinyin: Wu Yao Herbal drug name: Linderae Radix**	*Lindera aggregata* (Sims) Kosterm	Tender roots with yellowish white, powdery cross section, intense aroma	Dry-frying	*Lindera obtusiloba* Blume
				*Lindera aggregata* var. *aggregata*
				*Lindera umbellata* Thunb
**Pinyin: Yan Hu Suo Herbal drug name: Corydalis Rhizoma**	*Corydalis yanhusuo* W.T. Wang	Large, full, hard, and brittle pieces with light yellow, waxy-like cross section	Dry-frying, wine-frying, vinegar- or salt-frying of slices	*Corydalis decumbens* (Thunb.) Pers. *Corydalis glaucescens* Regel
				*Corydalis humosa* Migo
				*Corydalis turtschaninovii* Besser
				*Curcuma longa* L
**Pinyin: Chuan Xiong Herbal drug name: Chuanxiong Rhizoma**	*Ligusticum stratium* DC.	Large, fleshy, solid, and heavy rhizomes with an intense aroma and a bitter, acrid taste turning slightly sweet	Dry-frying, wine-frying of slices	*Ligusticum chuanxiong* cv. Fuxiong *I*mmature rhizomes sold cheaper as inferior material. Occasional adulteration/unofficial substitutes of rhizomes from varying plant species recorded in international trade
**Pinyin: Gan Cao Herbal drug name: Glycyrrhizae Radix**	*Glycyrrhiza inflata* Batalin *Glycyrrhiza uralensis Fisch*	Thin, tight, reddish-brown cork, solid and heavy cortex, yellowish white and powdery surface on cross section	Dry-fried, honey-prepared slices	Substitution/adulteration not common
	*Glycyrrhiza glabra L*			
**Pinyin: Xiang Fu Herbal drug name: Cyperi Rhizoma**	*Cyperus rotundus* L	Large, full, hard, and solid rhizomes with intense aroma	Processed (boiled with yellow rice wine/vinegar and dried in the sun, blackened (dry-fried or over heat until inside scorched yellow, outside black), dry-fired, four substance prepared (soaked and mixed with rice vinegar, yellow rice wine, cooked honey and salt/ginger juice/boy’s urine	*Cyperus stoloniferus* Retz
**Pinyin: Hong Hua Herbal drug name: Carthami Flos**	*Carthamus tinctorius* L	Long, dark, or fresh red soft flowers with intense aroma	Drying	Substitution/adulteration not common
**Pinyin: Zhi Ke Herbal drug name: Aurantii Fructus**	*Citrus* x *aurantium* L	Large fruit with greenish brown surface, hard soil texture, small thick pulp, fresh, aromatic fragrance	Dry-fried, charred fried (until scorched and blackened externally)	*Citrus maxima (Burm.) Merr*
				*Citrus medica* L
				*Citrus trifoliata* L

^a^ Information retrieved from Bensky et al. (2004); Scheid et al. (2015); Leon and Yu-Lin (2017)). Overlapping ingredients of the two formulae: Moutan Cortex, Paeoniae Radix Rubra, Persicae Semen are not repeated, see Table 1.

### High-performance thin-layer chromatography (HPTLC)

Fifty samples were prepared. Sample with the code *Cyperi Rhizoma 10* is not included. HPTLC marker compounds and other chemicals were obtained from Merck (Darmstadt, Germany) and Sigma-Aldrich (St. Louis, MA, United States), and botanical reference standards were obtained from ChemStrong Scientific, China. Marker compounds and botanical reference standards were prepared according to Hong Kong Chinese Materia Medica guidelines (HK Chinese Materia Medica, 2021) unless reported differently. A specific HPTLC method was used for each plant, see Supplementary Data S1. Samples (0.50 g) were dissolved in 10 mL of ethanol, followed by sonication for 20 min and filtration using Merck Millex PES syringe filters (0.22 μm). Each TLC plate (silica gel 60 F_254_ Merck, Darmstadt, Germany) was visualized under white light and UV 254 and 366 nm. Marker compounds, samples and herbal references were spotted in bands of 8.0 mm width, using a CAMAG Linomat 5 instrument (CAMAG, Muttenz, Switzerland). The development distance differed depending on the ingredient being detected, as indicated in each testing method (Supplementary Data S1) and is calculated from the lower edge of the plate using CAMAG Automatic Developing Chamber (ADC 2). For derivatization, CAMAG derivatizer was used when the derivatizing reagent complied with CAMAG’s guidelines, otherwise manual spraying was employed. Following development and derivatization, plates were visualized under white light, UV 254 nm and 366 nm using CAMAG’s Visualizer (Muttenz, Switzerland). All data was acquired and processed using VisionCATS 2.1 software (CAMAG, Muttenz, Switzerland).

For chemical fingerprints, all ingredients were named with herbal drug names. Each sample was compared to the fingerprint of the pharmacopeial botanical reference standards. To display the results, a band intensity score (BIS) with a scale from zero to five was visually assigned for all ingredients. Each band in the fingerprints was given a score from zero to five, based on the intensity perceived by the naked eye, compared to the standard, where zero is “not detectable”, and five is the highest intensity. BISs with scales 0-5 refer to the quality of the ingredients’ entire fingerprint with respect to the visibility and positions of bands for the pharmacopoeial reference marker compounds and botanical references in each chromatogram (Mück et al., 2023).

### Conventional barcoding with Sanger sequencing

The DNA extraction kit E.Z.N.A SP plant DNA kit (Omega Biotek, Norcross, United States of America) was used according to the manufacturer’s instructions except for a larger quantity of starting material (up to 30 mg) and an elongated lysis with larger volumes of buffer in all steps prior to DNA binding to HiBind columns (e.g., 1.6 mL SP1 buffer at 65°C for 1 h). Samples were mixed frequently during incubation and the final elution volume was 100 μL. Extracted DNA was quantified and polymerase chain reactions were performed to amplify the two internal transcribed spacer regions of the nuclear ribosomal RNA with primers based on ITS1_17SE_F and ITS1_5.8I_R and ITS2_5.8I_F and ITS2_26SE_R (Sun et al., 1994). Expected amplicon sizes were approximately 600 bp for nrITS1 and 100-200 bp for nrITS2. Polymerase chain reactions (PCR) were carried out in 12,5 μL reactions consisting of 2.5 μL of template DNA, 6.25 μL of AccuStart II PCR ToughMix (AccuStart, Quantabio, MA, United States of America), 0.16 µM of each, forward and reverse primer. The PCR cycling protocol consisted of initial denaturation at 94°C for 3 min, followed by 35 cycles of denaturation at 94°C for 10 s, annealing at 52°C for ITS1 and 59°C for ITS2 for 15 s, and elongation at 72°C for 1 min followed by a final elongation step at 72°C for 1 min. Gel electrophoresis was performed to check amplified DNA products. Trouble shooting was conducted to reduce amplification errors: For missing, - and double bands, DNA templates were diluted 50 times and annealing temperature was lowered for ITS1. PCR products were then treated with illustra ExoProStar 1-STEP (Cytvia, Marlborough, United States of America) with a modified protocol with 10 x dilution and 45 min incubation, and sent for Sanger sequencing (Macrogen, Amsterdam). Visualization and assessment of each obtained sequencing chromatogram was conducted using the software program Geneious by Dotmatics (Boston, MA, United States). Taxonomic assignment was performed via an optimized BLASTn search by selecting base calling score Q > 20 for unique top hits and verifying percent identity with a threshold >95% for identifications at genus level and 98% at species level. The results are presented in an overview of five categories, which include identification at species, genus and family level, such as unexpected identification of ingredients and failed identifications. All ingredients are listed with scientific binomials.

### Dual locus metabarcoding

DNA extraction, PCR, normalization and pooling of amplicons, such as library preparation and sequencing was conducted according to Mück et al. (2023). The samples were sequenced and analyzed alongside the sample set presented in Mück et al. (2023). We used dual index fusion primers for amplicon libraries of the internal transcribed spacers nrITS1 and nrITS2, based on 18S-ITS1F and 58S-ITS1R (Omelchenko et al., 2019), and ITS2F and ITSp4 primers (Timpano et al., 2020). PCRs were run using indexing primers as in Raclariu-Manolică et al. (2023b) with applying the indexing strategy of Fadrosh et al. (2014).

Bioinformatic processes related to the metabarcoding analysis were done as described in (Mück et al., 2023). After applying strict filtering controls to delete any false positive detections for each sample, the taxonomic assignment step was conducted by selecting top-scored species as the target species (search tool: BLAST) (Yao et al., 2022). OTUs were checked for species delineation with ASAP, assembling species by automatic partitioning (Puillandre et al., 2021) unique species were pooled together to avoid overinflation of the observed species range. In the final presentation of results, taxonomic identification hits of ingredients are categorized into expected substitutes, expected ingredients at genus and species level, and unexpected ingredients.

## Results

**FIGURE 1:**
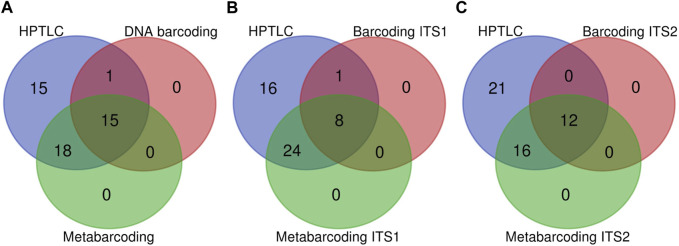
Three venn diagrams comparing HPTLC, DNA barcoding and DNA metabarcoding methods. **(A)** Pooled results for nrITS1 and nrITS2 for DNA barcoding and metabarcoding. **(B)** Comparison of identification hits for expected ingredients differentiating DNA barcoding nrITS1 and metabarcoding nrITS1. **(C)** Comparison of identification hits for expected ingredients differentiating DNA barcoding nrITS2 and metabarcoding nrITS2.

We performed a census for detected expected ingredient counts to compare the three methods. Here we pooled the results for nrITS1 and nrITS2 for DNA barcoding and for DNA metabarcoding, gaining a cumulative count which represents the performance of each method. Both identification hits at genus and species level were considered as expected ingredients. HPTLC analysis resulted in 49 positive identifications of expected ingredients, whereas traditional DNA barcoding yielded 16 positive identifications and DNA metabarcoding 33 (See Figure 1A.). In detail, with HPTLC, all ingredients were identified except for one sample of Linderae Radix (Linderae Radix 1), which was not identified across either of the methods. With DNA barcoding ten expected ingredients were identified at species level and another six at genus level. With metabarcoding four expected ingredients were identified at species level, including one, Linderae Radix (sample: Linderae Radix 3), that was not identified with DNA barcoding. Looking at identification hits for expected ingredients using DNA barcoding nrITS1 and metabarcoding nrITS1 separately, five expected ingredients were identified at species level with DNA barcoding nrITS1 and one at species level with metabarcoding nrITS1 (Figure 1B). Looking at identification hits for expected ingredients via DNA barcoding nrITS2 six expected ingredients were identified at species level, and three at species level with metabarcoding nrITS2 (Figure 1C) (For more information see Supplementary Data S2).

### HPTLC

**FIGURE 2:**
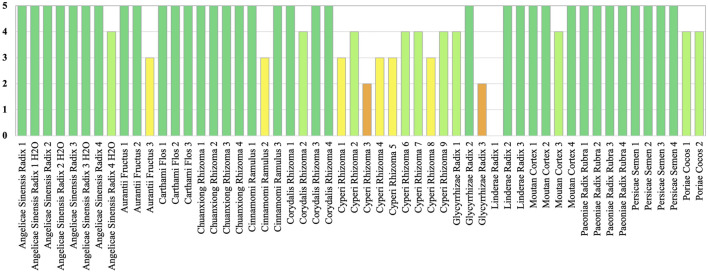
HPTLC results are visualized with band intensity scores (BIS, 0-5) for level of identification.

The band positions and visibility of the chemical markers of all ingredients appear with characteristic colors and Rf values. All botanical reference materials show clear chromatograms and all marker compounds were identified (Supplementary Data S3). With HPTLC, we obtained 49 positive identifications for expected ingredients across the sample range. One sample with the ingredient of Linderae Radix was not positively identified (see Figure 2). Ingredients refer to the accepted species under the ingredients as listed in (Chinese Pharmacopoeia Committee, 2020).

### DNA barcoding with nrITS1 and nrITS2

**FIGURE 3:**
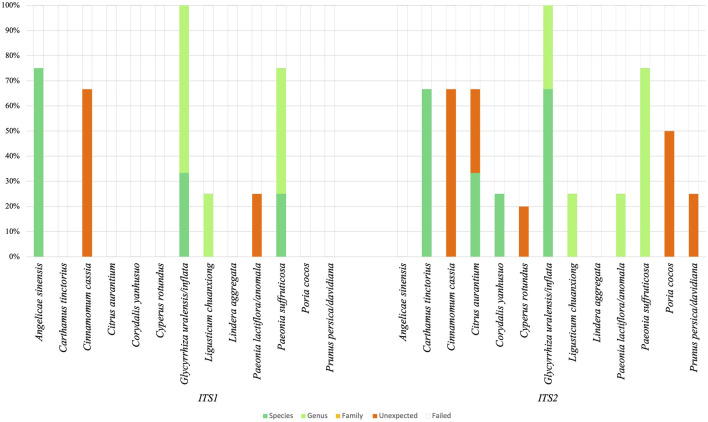
DNA barcoding results are presented in an overview of five categories: Identification at species (dark green), genus (light green) and family level (yellow), unexpected identifications (orange) and failed identifications (remainder). Ingredients are listed with scientific binomials.

Using traditional identification via DNA barcoding, sample analysis with nrITS1 resulted in nine positive identification hits for the target ingredient representing the sample. The analysis for nrITS2 resulted in 12 positive identification hits (See Supplementary Data S2). Five target ingredients were authenticated at species level with nrITS1, i.e., *Paeonia suffruticosa* (ingredient codes: Moutan Cortex 2), *Glycyrrhiza uralensis/inflata* (Glycyrrhizae Radix 3) and *Angelica sinensis* (Angelica Sinensis Radix 1, 2, 4). Furthermore, we identified *Paeonia suffruticosa* (Moutan Cortex 4), *G. uralensis/inflata* (Glycyrrhizae Radix 1 and 3) and *Ligusticum chuanxiong* (Chuanxiong Rhizoma 4) at genus level. The remaining ingredients yielded unexpected identification hits or failed completely due to poor amplification of primers nrITS1 or poor sequencing chromatograms. We identified six ingredients at species level with nrITS2, i.e., *G. uralensis/inflata* (Glycyrrhizae Radix 2, 3), *Corydalis yanhusuo* (Corydalis Rhizoma 2), *Citrus aurantium* (Aurantii Fructus 2), and *Carthamus tinctorius* (Carthami Flos 2 and 3). Six ingredients could furthermore be identified at genus level: *G. uralensis/inflata* (Glycyrrhizae Radix 1), *L. chuanxiong* (Chuanxiong Rhizoma 4), *Paeonia lactiflora/anomala* (Paeoniae Radix Rubrae 3), and *Paeonia suffruticosa* (Moutan Cortex 1, 2 and 3). In contrast to identification with nrITS1, with nrITS2 no ingredients were identified for *A. sinensis*. For both markers, identifications of ingredients with *Lindera aggregata* (Linderae Radix) failed. Results are illustrated in Figure 3.

### Metabarcoding with nrITS1 and nrITS2

A dataset with 2 258 042 reads was obtained for nrITS1 with an average of 13,685 reads per sample. Respectively, a dataset with 4 873, 864 reads was obtained for nrITS2 with an average of 29,361 reads per sample. For nrITS1, one sample failed to pass the bioinformatic trimming and filtering criteria, for nrITS2 four samples did not pass the criteria and these samples were excluded from the final results. Operational taxonomic units (OTUs) could be assigned for all samples for markers nrITS1 and nrITS2. The raw dataset of nrITS1 contained 508 OTUs, and after applying strict quality selection criteria and pooling 73 unique species were identified. The raw dataset of nrITS2 contained 347 OTUs and 53 species were identified after applying the quality criteria. The sample analysis using nrITS1 resulted in 18 samples with only unexpected identification hits and 32 samples with at least one positive identification hit for the expected ingredient. The analysis using nrITS2 resulted in 22 samples with only unexpected identifications and 28 samples with at least one positive identification hit (See Supplementary Data S2).

**FIGURE 4:**
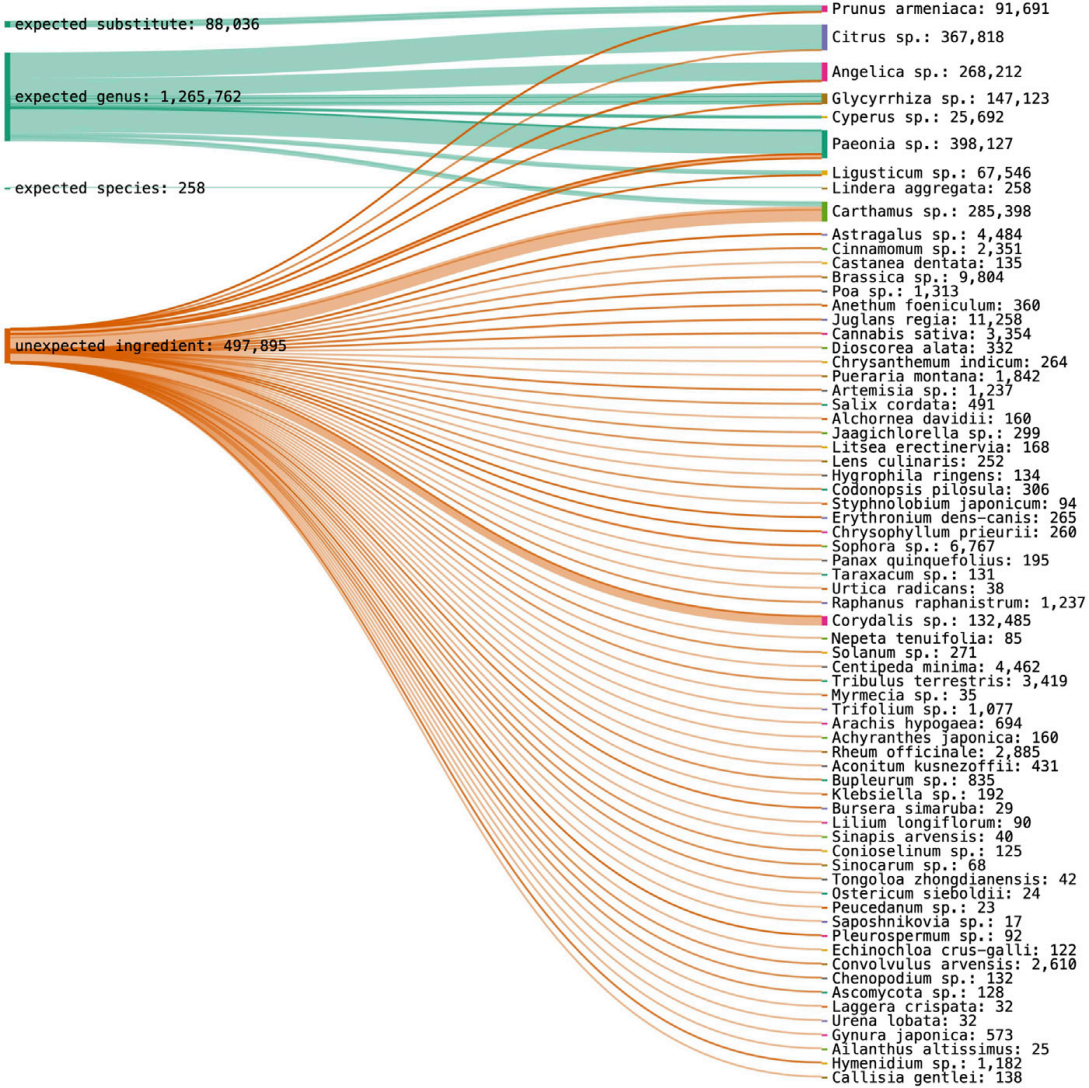
DNA metabarcoding results for nrITS1 grouped by identification class (expected substitute, expected genus, expected species, unexpected ingredient) combined with the detected species richness in read abundance.

Using nrITS1, ten expected plant taxa and one substitute species could be identified, while three target ingredients were not detected. Proportionally to detected species abundance in one sample, 14.8% of Paeoniae Radix Rubra could be identified at genus level, 26.7% of Moutan Cortex at genus level, 5.6% of Linderae Radix at species level, 50% of Glycyrrhizae Radix at genus level, 8.3% of Cyperi Rhizoma at genus level, 11.1% of Chuanxiong Rhizoma at genus level, 20% of Carthami Flos at genus level, 16.7% of Aurantii Fructus at genus level and 21.4% of Angelica Sinensis Radix at genus level. Fifty-seven percent of Persicae Semen was identified as a substitute species. Poriae Cocos, Corydalis Rhizoma and Cinnamomi Ramulus were not detected in their corresponding samples. The results for nrITS1 are visualized with the total species abundance across all samples in Figure 4.

**FIGURE 5:**
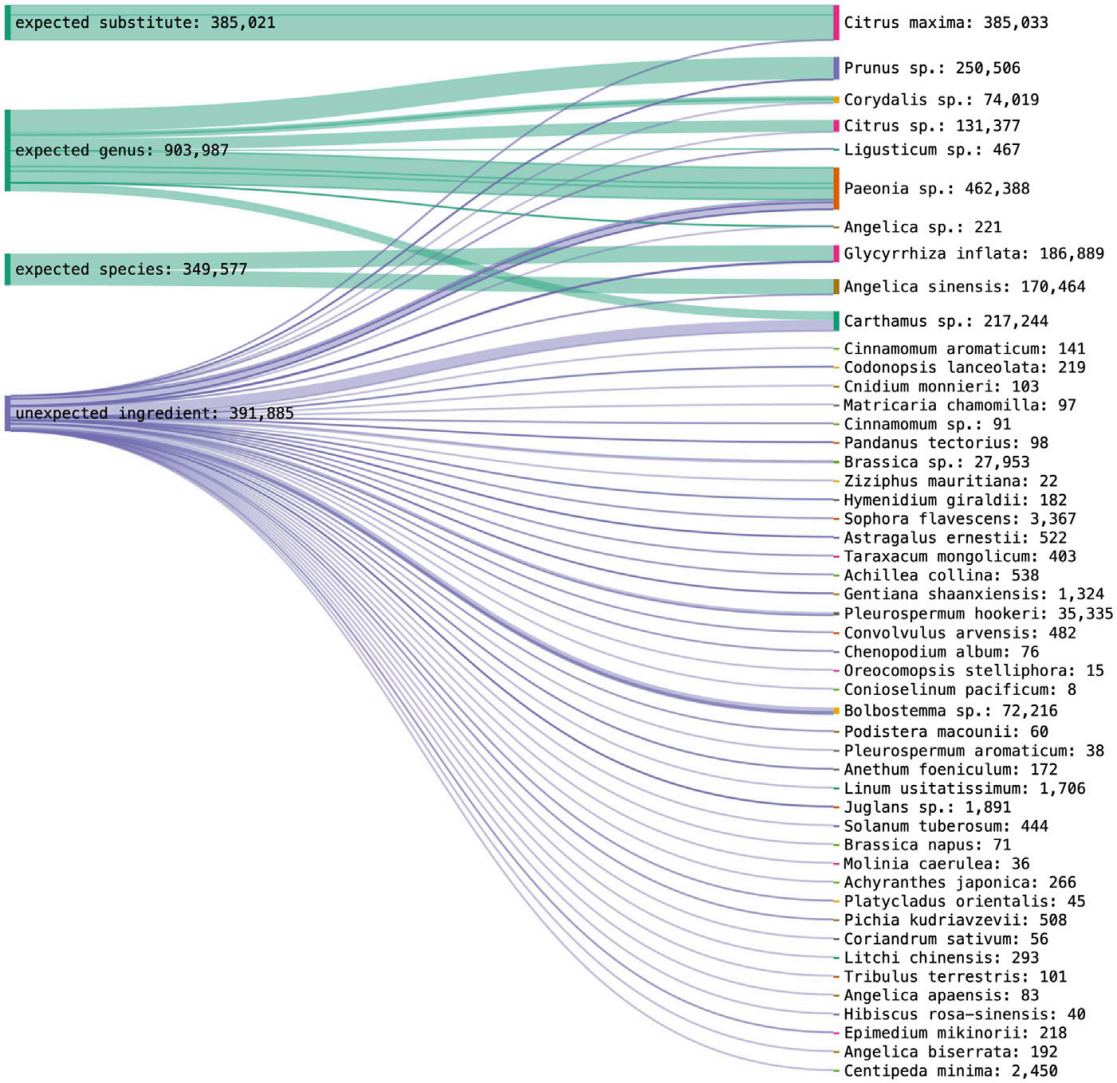
DNA metabarcoding results for nrITS2 grouped by identification class (expected substitute, expected genus, expected species, unexpected ingredient) combined with the detected species richness in read abundance.

Using nrITS2, nine target ingredients and one substitute species could be identified, while four target ingredients were not detected. More specifically, each could be identified at genus level at the following percentages, Persicae Semen (63%), Paeoniae Radix Rubra (10.1%), Moutan Cortex (40%), Glycyrrhizae Radix (75%), Corydalis Rhizoma (17.7%), Carthami Flos (33.3%), and Aurantii Fructus (10%). Angelica Sinensis Radix could be identified 20% at genus level and 20% at species level, and Chuanxiong Rhizoma 15.4% at genus level and 7.7% at species level. Aurantii Fructus was revealed as an expected substitute in 5% of Aurantii samples. Poriae Cocos, Linderae Radix, Cyperi Rhizoma and Cinnamomi Ramulus were not detected in their corresponding samples. The results for nrITS1 are visualized with the total species abundance across all samples in Figure 5.

## Discussion

Herbal medicines and dietary supplements, as well as their raw ingredients, pose a variety of challenges for quality control. Most pharmacopoeias are focused on analytical chemical methods for quality control based on detection of target compounds. More recent studies have shown that other chemical approaches yield more detailed and complementary insights, which also allow for the detection of contaminants and adulterants at low levels. The development of complex chromatographic, spectroscopic and hyphenated methods has contributed to the expansion and development of pharmacopoeial monographs, and responded to regulatory demands and expectations of herbal quality on the market (Fitzgerald et al., 2019). HPTLC is the most advanced and robust form of thin-layer chromatography (TLC) in applied herbal quality control (Velho-Pereira et al., 2011; Nicoletti et al., 2013; Booker et al., 2016; 2014; Nandanwadkar et al., 2016; Raclariu et al., 2018; Kandil et al., 2022). It offers good resolution, detection sensitivity, and enhanced *in situ* densitometric quantification compared to conventional TLC (Ram et al., 2011; Sonia, 2017; Bhusan Champati et al., 2023) and can be applied simultaneously in an efficient and economical manner (Kunle, 2012; Sonia, 2017; Kandil et al., 2022). Furthermore, emerging research trends are centered around gas chromatography (GC), mass spectroscopy (MS), UV/visible spectrophotometric techniques, nuclear magnetic resonance (NMR) and tandem approaches (Booker et al., 2014; 2016; Liang et al., 2018), which allow for a more complex analysis and characterization of single compounds into parts per billion range (MS) and give detailed fingerprints of metabolites across varying polarities (NMR) (Fitzgerald et al., 2019). Recent studies furthermore propose a combination of multidimensional chromatography with chemometric methods, which exhibit stronger capacity for screening and separating bioactive compounds in complex TCM samples (Yang et al., 2023). For the detection of authenticity issues and compound adulteration, specifically chromatography, its tandem technologies and combinations with exploration/classification/regression algorithms, are relatively mature and widely applied (Liu et al., 2023). Additionally, innovative approaches emerge from modern analytical approaches, such as metabolomics (Heinrich, 2015; Emwas et al., 2019; Xiao et al., 2022; Nikolaichuk et al., 2023). Furthermore, new possibilities arise for the establishment of a more elaborate herbal quality control system by developing quality markers for comprehensive fingerprint and multicomponent analysis and novel standardization practices of herbal materials (Zhang, 2016; Liu et al., 2023; 2018; 2017; Liu, 2017; Yang et al., 2017; Bai et al., 2018; Li et al., 2019; He and Zhou, 2021; Noviana et al., 2022; Kaggwa et al., 2023).

**FIGURE 6:**
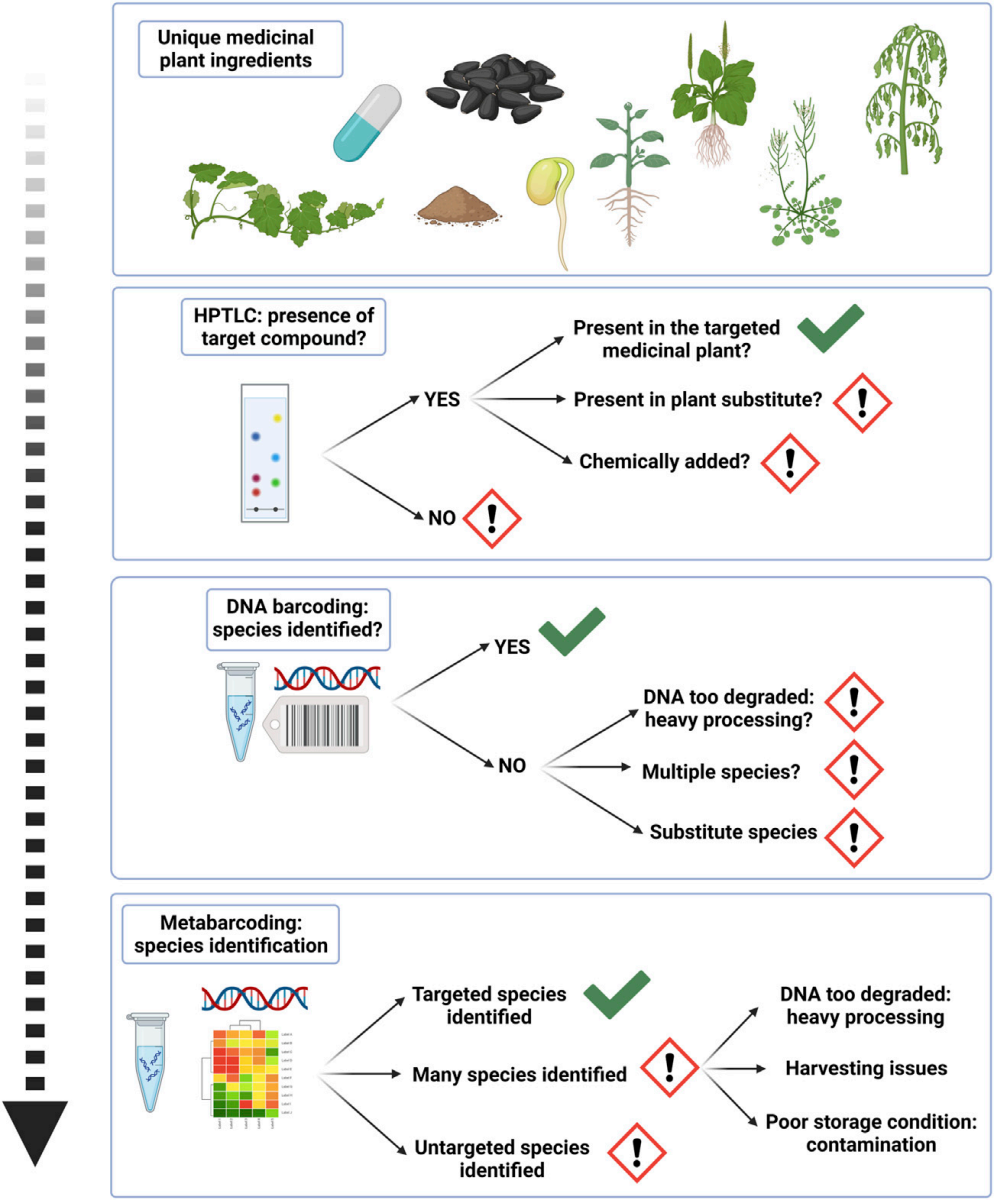
A tiered authentication strategy for processed herbal ingredients yields cumulative insight in ingredient quality.

In conjunction with chemical fingerprinting, researchers have gradually turned their focus to DNA based molecular methods, which are recognized as techniques to identify edible and medicinal plant species, and also to detect their substitutes and adulterants in crude or processed products (Raclariu et al., 2017; Raclariu-Manolică et al., 2023b). These methods are independent of species life stage, tissue type, or the physiological conditions of constituents (Techen et al., 2014; Lo and Shaw, 2018b; Hao and Xiao, 2020) and can discriminate botanicals at species level (Besse et al., 2021; Mishra et al., 2016). As such, DNA barcoding is used to qualitatively authenticate herbal medicines by validating the identity of the corresponding species in industrial quality control procedures (Raclariu et al., 2018; Zhu et al., 2022). With most traditional methods, it is difficult to identify crude herbal drug material on species level, but with the help of DNA barcoding, pharmacopoeial monographs of many medicinal plant species could be advanced for accurate, reliable and effective species identification (Song et al., 2009; Chen et al., 2017; Zhu et al., 2022). Conventional DNA barcoding has been adopted by various national pharmacopeias (Chen et al., 2017). User friendly and accessible tools have emerged for correct species assignment with DNA barcoding alongside its establishment as a regulatory post quality control method for herbals, like the Medicinal Materials DNA Barcode Database (MMDBD) (Wong et al., 2018). However DNA barcoding is exclusively fit for unprocessed and single species plant materials, which have not been exposed to processing techniques resulting in DNA degradation (Raclariu et al., 2018). Evolving high-throughput sequencing techniques, like DNA metabarcoding overcome limitations of conventional DNA barcoding and are used for investigation of total species diversity and non-targeted species in processed herbal products (Arulandhu et al., 2017; Raclariu et al., 2018; Omelchenko et al., 2019; Seethapathy et al., 2019; Anthoons et al., 2021; Raclariu-Manolică and de Boer, 2022; Raclariu-Manolică et al., 2023a; Mück et al., 2023). More recent genomics approaches, including genome skimming and shotgun metagenomics, have the potential to overcome limitations of PCR-based methods, like PCR biases due to primer mismatch, limited number of applicable barcodes, limited DNA degradation, and are expected to yield higher discriminatory power (Wu and Shaw, 2022; Raclariu-Manolică, 2023a). High operational costs currently limit wider application of genomics approaches (Manzanilla et al., 2022), and to date only a few metagenomic studies have been conducted in the field of herbal authentication (Xin et al., 2018; Handy et al., 2021). Xin et al. (2018) performed shotgun sequencing of CHMs to obtain the barcode regions ITS2, psbA-trnH, and matK. Barcoding techniques can further be used in conjunction with metabolomics, transcriptomics or proteomics (Mishra et al., 2016; Raclariu-Manolică et al., 2023b). The possibility to process data via multivariate analysis, pattern recognition and metabolomics then gives a broader scope for applications in medicinal plant analysis and the spectrum of compounds found within medicinal plants (Feng et al., 2018; Nöst et al., 2019; Kaigongi et al., 2020; Raclariu-Manolică et al., 2023b). Modern analytical technologies combined with chemometrics are increasingly used for quality monitoring of medicinal plant matrices, but it is still challenging to choose the adequate type of analysis and statistical method as this is highly dependent on the specific authenticity issue (Liu et al., 2023). Besides, advanced genetic and chemical methods often require high analytical skills, are time consuming and expensive, are not always applicable to all natural compounds or biological materials and may not be suitable for general quality control. Since different quality control methods for herbal products yield different information, an integrated and practical authentication strategy is needed. We find that by establishing a multi-tiered quality control strategy using chemical and genetic methods, such as HPTLC, followed by DNA barcoding and metabarcoding, authentication procedures of processed herbal ingredients can be optimized (Li et al., 2017; Raclariu et al., 2017; Raclariu et al., 2018). First, chemical fingerprinting verifies the presence or absence of analytical marker compounds in sample materials and sheds light on the overall authenticity of plant materials. By evaluating and displaying a band intensity score (BIS) for chromatographically analyzed herbs (Mück et al., 2023), we can furthermore speculate on qualitative information such as cultivation and storage conditions, as chromatographic fingerprinting technique is useful for the evaluation of authentication, quality and investigation of consistency and stability of herbal drugs and can be useful at all stages of the herbal supply chain (Liang et al., 2010; Raclariu et al., 2017). It enables a qualitative profile, such as detection of low-quality aspects of phytochemical contents and significant product-to-product variation (Ichim and Booker, 2021). This is an important consideration for applying authentication procedures, as adulteration and substitution may occur by means of using other, cheaper plant parts (Ichim and de Boer, 2020). By virtue of choosing HPTLC as the primary selective method for a tiered authentication strategy, we optimize inclusion of all samples, including those that have lost their diagnostic microscopic characteristics, or where DNA cannot be recovered (Ichim and Booker, 2021). At the second tier, DNA barcoding reveals the presence or absence of target DNA, and provides a definite answer on the possibility of identification via the quality of genetic sequencing chromatograms (Patel et al., 2018; Raclariu et al., 2018). Thus, it indirectly gives information on the level of processing of herbal materials. At the third tier, with the help of metabarcoding more qualitative aspects can be inferred and to help with creating transparency along the supply chain of herbal products. Detected species diversity with metabarcoding yields an important insight, and the potential to check the integrity of plant ingredients and receive an approximation for qualitative information, like harvesting, storage and processing conditions, as well as conservation issues around wild harvesting of medicinal plants (Staats et al., 2016; Arulandhu et al., 2017; Raclariu et al., 2017; Raclariu et al., 2018; Seethapathy et al., 2019; Turon et al., 2019; Anthoons et al., 2021; Raclariu-Manolică et al., 2023a) (See Figure 6).

Similar applied strategies combining chromatography and DNA barcoding technique for herbal authentication have been previously assessed. A study by Raclariu et al. (2017) shows that HPTLC can be efficiently applied for the detection of target compounds in *Echinacea* products, while DNA metabarcoding complements the analysis by detecting non-targeted species in these herbal products and gives information on species not listed as ingredients. In another study by Seethapathy et al. (2018), DNA barcoding was coupled with NMR and suggested as a regulatory tool for the authentication of *Garcinia* fruit rinds and food supplements. While DNA barcoding gives information on the level of adulteration, NMR provides quantitative information on target chemical constituents. Handy et al. (2021) applied DNA metabarcoding and genome skimming, coupled with HPLC–UV analysis in a more advanced approach to assess 20 dietary supplements of Echinacea. Genome skimming was found to be more effective than DNA metabarcoding for species-level authentication within the Echinacea genus and might be used instead of metabarcoding once its application is more economical for applied herbal quality control. The trend in TCM chemical quality control is towards establishing chemometric applications based on the data gathered from different quality control methods (Liu et al., 2023; Yang et al., 2023). A review by Kandil et al. (2022) on advances in quality control of fenugreek seeds, highlights that chromatography, like HPTLC and DNA-based methods, like DNA barcoding and the NGS when coupled to multivariate analysis, can yield promising results in herbal quality control.

This proposal for a multi-tiered quality control strategy using chemical and genetic methods, such as HPTLC followed by DNA barcoding and metabarcoding, allows for the integration of multifaceted information on the quality of diverse herbal medicine matrices, like CHMs. Its advantage and novelty lies in the accessibility and informative resolution for the applied and regulatory sector. By combining these methods, we take a stride towards establishing an herbal quality control system, which can be further enhanced by developing multivariate indices assessing the combination of analytical outcomes.

In this study, by applying a band intensity score (BIS) (Mück et al., 2023), we were able to grade qualitative characteristics and to differentiate between the quality of identification amongst ingredients with a scale from zero to five (see Figure 2). The variation determination of common analytes in the set of chromatographic fingerprints could provide useful qualitative and quantitative information on the characteristic components of herbal medicines investigated. Nevertheless many analytical chemistry based methods are sensitive to fraud through adulteration of ingredients (Gafner et al., 2023). Furthermore, HPTLC meets challenges when comparing a number of botanicals from different source materials and unilateral standardization of methods for TCM preparations is difficult. This is because concentrations for chemical marker compounds can be different for botanical materials of varying origin and natural fluctuations in chemical compounds can occur for different growth cycles, eco-regions, and times of the year (Yamamoto, 1988; Tobyn et al., 2011). Besides, TCM ingredients can originate from varying accepted plant species, whilst the standard identification method in the pharmacopeia remains the same for either species (Mück et al., 2023). Moreover, the varying processing techniques of CHMs can be different for ingredients of the same plant species and may alter the chemistry of compounds (Wang et al., 2019) (see Tables 1, 2).

Authentication via DNA barcoding resulted in poor identification for most samples. This could be due to the processing state of sample materials, e.g., processing of decoction material resulted in contamination with other plant material and extensive DNA degradation. DNA target amplification (Sun et al., 1994) yielded poor results with messy and or overlaying sequencing chromatograms (Patel et al., 2018). NrITS2 performed better than nrITS1 for expected identification hits via Sanger sequencing and for metabarcoding in terms of expected species hits with number of taxon assigned OTUs. The internal transcribed spacer region ITS2 is known as an efficient sequence for taxa identification in comparison to the full-length ITS and has been extensively used for the identification of medicinal plants (Gao et al., 2010; Han et al., 2013). Both ITS1 and ITS2 can provide comprehensive species identification for molecular analysis of TCMs at lower taxa levels (Chen et al., 2017; Yao et al., 2022; Zhu et al., 2022). The combination of nrITS1 and nrITS2 may be used in a cumulative approach to receive enhanced ingredient information (Zhu et al., 2022). Interestingly, for metabarcoding with nrITS1 the genus *Cyperus* could be identified, while it was not detectable with nrITS2. With nrITS2 we could identify two ingredients, *A. sinensis* (Oliv.) Diels and *Glycyrrhiza inflata* Batalin, while with nrITS1, we identified *L. aggregata* (Sims) Kosterm. The species abundance of unexpected ingredients varied between nrITS1 and nrITS2. On another note, this highlights that biological DNA-based assessments are highly dependent on well-curated nucleotide sequence repositories (Taberlet et al., 2007; Howard et al., 2020). Shortcomings of barcoding exist due to gaps in reference databases for DNA markers (Zhu et al., 2022) and challenges with delimitating species through delimitation models (Taylor and Harris, 2012; Howard et al., 2020; Puillandre et al., 2021). Another methodological challenge for DNA-based identifications are several plant compounds including polysaccharides, polyphenols, lipids, essential oils, alkaloids and other secondary metabolites frequently found in medicinal plant species and their processed counter parts, which can interfere with DNA extraction and PCR amplification (Porebski et al., 1997; Shepherd and McLay, 2011; Sudan et al., 2017). Interference from those compounds and processing techniques of source material can lead to false-negative results (Mück et al., 2023). Thus, well-established DNA extraction procedures are crucial when dealing with complex, poly-herbal samples (Corrado, 2016; Lo and Shaw, 2018a; 2018b). Medicine processing techniques, like traditional *Pao Zhi* in TCM, then affects the DNA quality drastically and is a common cause for highly degraded DNA in CHMs. In detail, *Pao Zhi* affect the DNA quality through processes like roasting, baking, stir-frying, and the application of liquid or solid excipients (Engelhardt et al., 2018; Wu et al., 2018) (see Tables 1, 2). Hence, we assume that the main trade-offs for molecular authentication of TCMs are degraded and fragmented DNA, which cannot be amplified and assessed with the common barcoding primers for ITS1 or ITS2. Shorter mini-barcodes can lack the discriminatory power to identify samples on species level (Di Bernardo et al., 2007; Lo and Shaw, 2019). Our results also suggest false-positive reads from minimal contaminations of other species, which is common in pharmacy preparation rooms and usually don’t have a negative impact on quality, safety, and efficacy of the ingredients (Mück et al., 2023). Monographs on “herbal drugs” of the European Pharmacopoeia allow for up to 2% of foreign matter unless differently stated in a specific herb monograph (EMA, 2006; EMA, 2011). Overall, DNA metabarcoding is limited by the quality, processing state, or product type of isolated material, the DNA purification procedure, primer choice, amplification procedure, library preparation, sequencing technique, bioinformatic filtering and qualitative and clustering thresholds (Raclariu-Manolică, 2023a).

## Conclusion

Different authentication methods yield different insights into CHM quality. HPTLC is very useful for identification of individual CHM ingredients and was shown to be less affected by heavy processing techniques commonly applied in TCM. DNA barcoding is a suitable method for the identification of raw botanical materials prior to processing, but not equally applicable in assessing processed ingredients. DNA metabarcoding can be used for the authentication of herbal end products, post-marketing control and pharmacovigilance, and determining species composition in botanical medicines, such as TCMs, but yields positives that are hard to interpret without quantitative data. Current authentication, standardization and quality control procedures for herbal products and TCM preparations have shortcomings in inferring aspects of safety, purity and efficacy. In turn, we show that a tiered quality control strategy via HPTLC, followed by DNA barcoding and metabarcoding yields cumulative insights and overcomes limitations of each method. The diversity of standards on scope, requirements, definition and terminology of dietary supplement and herbal medicine categories is a strong argument for transparent science-based quality standards across regulations to increase quality along the growing supply chain. Herbal authentication needs to be expanded based on the standardization and verification of the entire framework for herbal quality control. Advancing and evolving conventional and emerging safety and quality assessment methods for herbal preparations is in the strong interest for both consumers, producers and regulators. A future perspective in TCM quality control may lead to advanced functional network pharmacology studies, where multi-omics, chemical information analysis, data-mining, and network toxicology are included.

## Data Availability

The data presented in the study are deposited in Zenodo repository, accession numbers: https://zenodo.org/doi/10.5281/zenodo.10204233, https://zenodo.org/doi/10.5281/zenodo.10204282, https://zenodo.org/doi/10.5281/zenodo.10204309, https://zenodo.org/doi/10.5281/zenodo.10204326.
