# Supplementary material for: Three-tiered authentication of herbal traditional Chinese medicine ingredients used in women’s health provides progressive qualitative and quantitative insight
Source: Front Pharmacol. 2024 Feb 5;15:1353434. doi: 10.3389/fphar.2024.1353434 (PMC10875096; doi:10.3389/fphar.2024.1353434)
Supplement: Supplementary file 1 [file DataSheet2.PDF]

Supplementary Data S2. Comparison of results obtained from HPTLC, DNA barcoding and metabarcoding for expected ingredients

Cumulative Identification Hits Σ	16		33		49
**Ingredients**	**ITS1 Sanger**	**ITS2 Sanger**	**ITS1 Metab**	**ITS2 Metab**	**HPTLC**
Poriae Cocos 2	N/A	N/A	✗	✗	✓
Poriae Cocos 1	N/A	✗	✗	✗	✓
Persicae Semen 4	N/A	N/A	✓	✓	✓
Persicae Semen 3	N/A	✗	✓	✓	✓
Persicae Semen 2	N/A	N/A	✓	✓	✓
Persicae Semen 1	N/A	N/A	✓	✓	✓
Paeoniae Radix Rubra 4	✗	N/A	✓	✓	✓
Paeoniae Radix Rubra 3	N/A	✓	✓	✓	✓
Paeoniae Radix Rubra 2	N/A	N/A	✓	✓	✓
Paeoniae Radix Rubra 1	N/A	N/A	✓	✓	✓
Moutan Cortex 4	✓	N/A	✓	✓	✓
Moutan Cortex 3	N/A	✓	✓	✓	✓
Moutan Cortex 2	✓✓	✓	✓	✓	✓
Moutan Cortex 1	N/A	✓	✓	✓	✓
Linderae Radix 3	N/A	N/A	✓✓	✗	✓
Linderae Radix 2	N/A	N/A	✗	✗	✓
Linderae Radix 1	N/A	N/A	✗	✗	✗
Glycyrrhizae Radix 3	✓✓	✓✓	✓	✓✓	✓
Glycyrrhizae Radix 2	✓	✓✓	✓	✓	✓
Glycyrrhizae Radix 1	✓	✓	✓	✓	✓
Cyperi Rhizoma 10 (not included in HPTLC)	N/A	N/A	✗	✗	N/A
Cyperi Rhizoma 9	N/A	N/A	✗	✗	✓
Cyperi Rhizoma 8	N/A	N/A	✗	N/A	✓
Cyperi Rhizoma 7	N/A	✗	N/A	✗	✓
Cyperi Rhizoma 6	N/A	N/A	✗	N/A	✓
Cyperi Rhizoma 5	N/A	N/A	✓	✗	✓
Cyperi Rhizoma 4	N/A	N/A	✓	✗	✓
Cyperi Rhizoma 3	N/A	N/A	✗	✗	✓
Cyperi Rhizoma 2	N/A	N/A	✗	✗	✓
Cyperi Rhizoma 1	N/A	✗	✓	✗	✓
Corydalis Rhizoma 4	N/A	N/A	✓	✓	✓
Corydalis Rhizoma 3	N/A	N/A	✓	✓	✓
Corydalis Rhizoma 2	N/A	✓✓	✗	✓	✓
Corydalis Rhizoma 1	N/A	N/A	✓	✓	✓
Cinnamomi Ramulus 3	N/A	N/A	✗	✗	✓
Cinnamomi Ramulus 2	✗	✗	✗	✗	✓
Cinnamomi Ramulus 1	✗	✗	✗	✗	✓

Chuanxiong Rhizoma 4	✓	✓	✓	✓	✓
Chuanxiong Rhizoma 3	N/A	N/A	✓	✓	✓
Chuanxiong Rhizoma 2	N/A	N/A	×	×	✓
Chuanxiong Rhizoma 1	N/A	N/A	×	N/A	✓
Carthami Flos 3	N/A	✓✓	✓	✓	✓
Carthami Flos 2	N/A	✓✓	✓	✓	✓
Carthami Flos 1	N/A	N/A	✓	×	✓
Auranti Fructus 3	N/A	N/A	✓	✓	✓
Auranti Fructus 2	N/A	✓✓	✓	✓	✓
Auranti Fructus 1	N/A	×	×	×	✓
Angelicae Sinensis Radix 4	✓✓	N/A	✓	✓✓	✓
Angelicae Sinensis Radix 3	N/A	N/A	✓	✓	✓
Angelicae Sinensis Radix 2	✓✓	N/A	✓	✓✓	✓
Angelicae Sinensis Radix 1	✓✓	N/A	×	N/A	✓

✓✓ identification on species level
✓ identification on genus level
× unexpected ingredient hit
N/A no identification possible
