# Supplementary material for: Three-tiered authentication of herbal traditional Chinese medicine ingredients used in women’s health provides progressive qualitative and quantitative insight
Source: Front Pharmacol. 2024 Feb 5;15:1353434. doi: 10.3389/fphar.2024.1353434 (PMC10875096; doi:10.3389/fphar.2024.1353434)
Supplement: Supplementary file 3 [file DataSheet3.PDF]

Supplementary Data S3. HPTLC chromatograms per ingredient (A.-M.)

A. Cinnamomi Ramulus
B. Poriae Cocos
C. Moutan Coretx and Paeoniae Radix Rubra
D. Persicae Semen
E. Angelicae Sinensis Radix
F. Angelicae Sinensis Radix H2O
G. Linderae Radix
H. Corydalis Rhizoma
I. Glyzyrrhizae Radix
J. Cyperi Rhizoma
K. Chuanxiong Rhizoma
L. Carthami Flos
M. Aurantii Fructus

See also Supplementary Table S1. A.-M.
- HPTLC methods

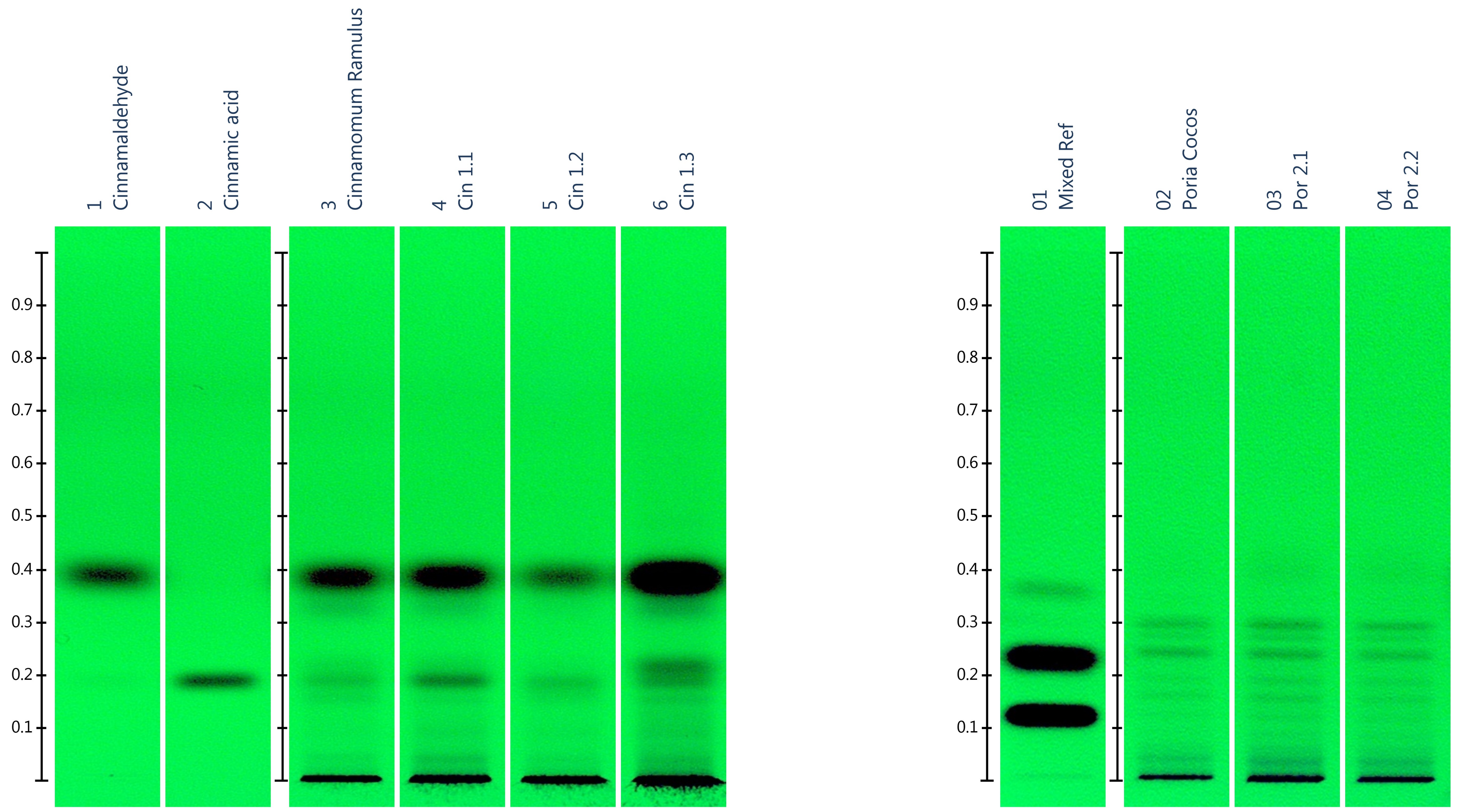

A. Cinnamomi Ramulus

B. Poriae Cocos

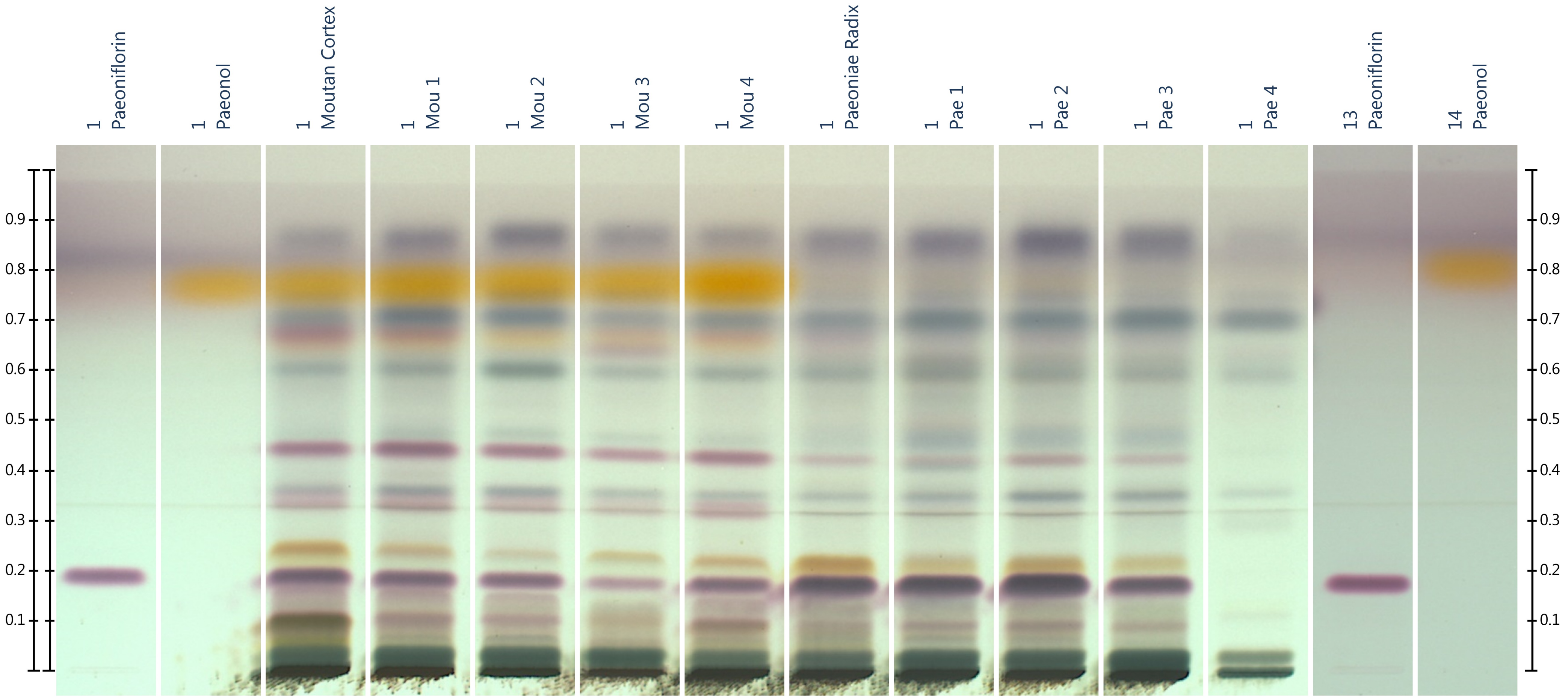

C. Moutan Cortex (1-4) and Paeoniae Radix Rubra (1-4)

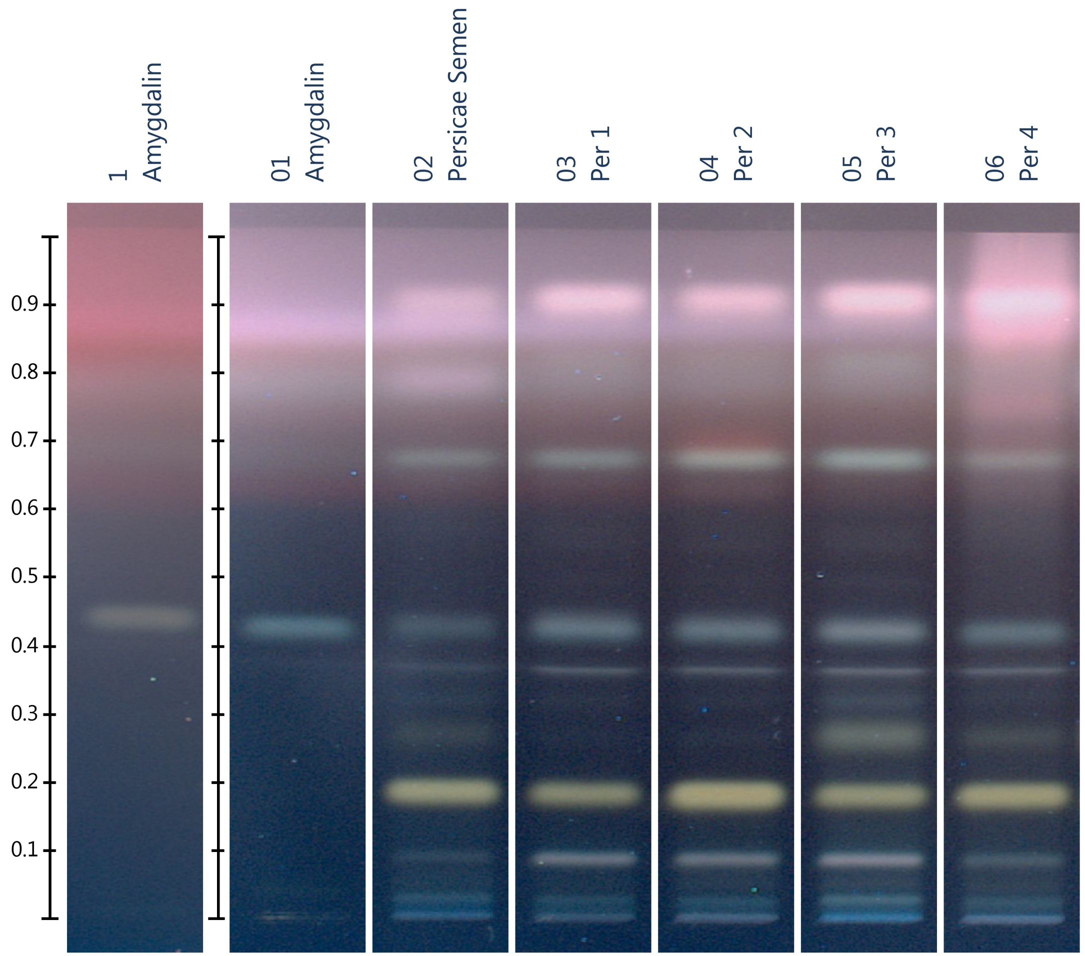

D. Persicae Semen

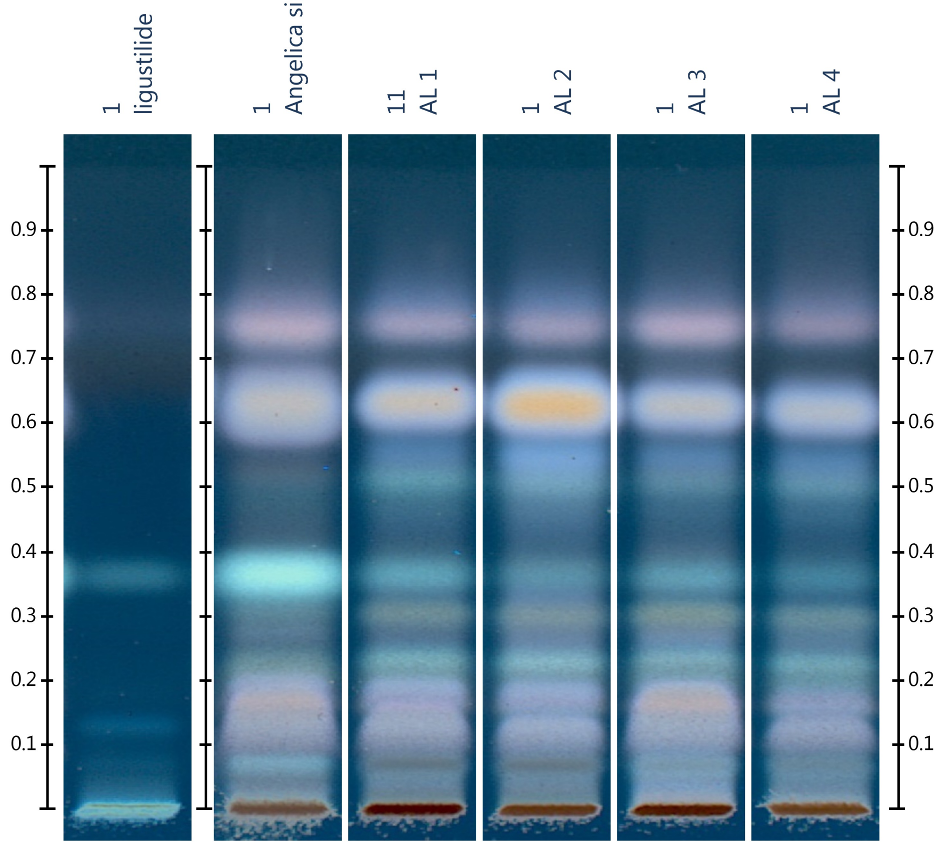

E. Angelicae Sinensis Radix

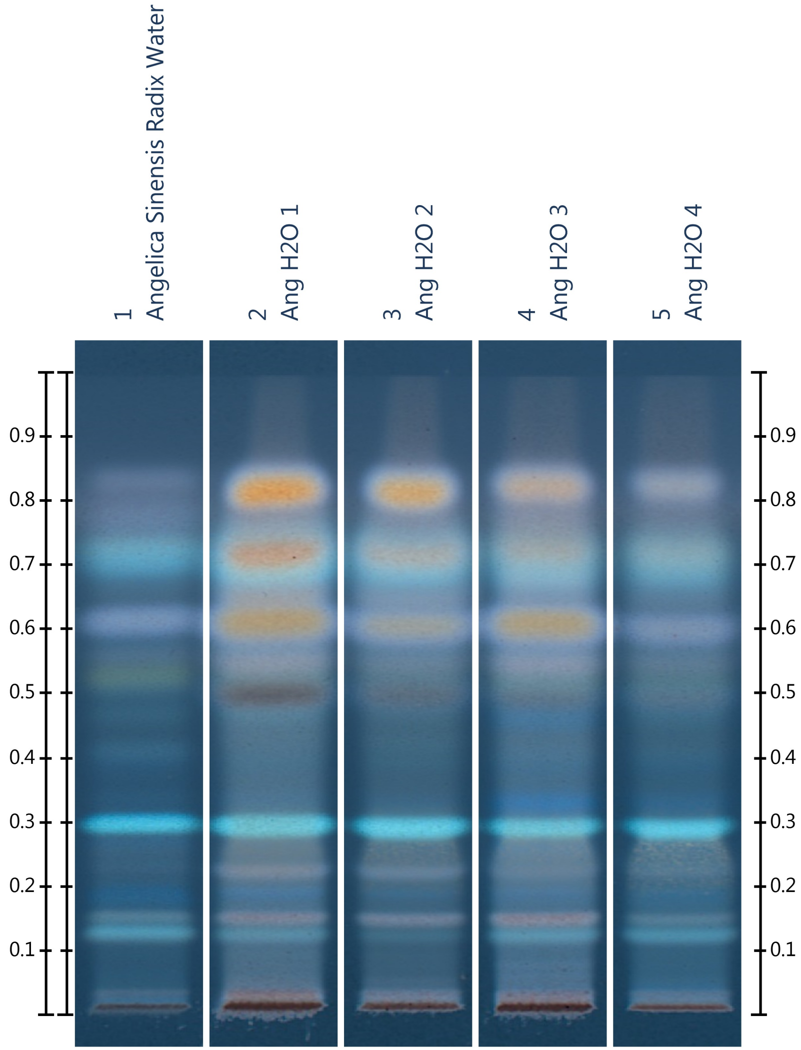

F. Angelicae Sinensis Radix H2O

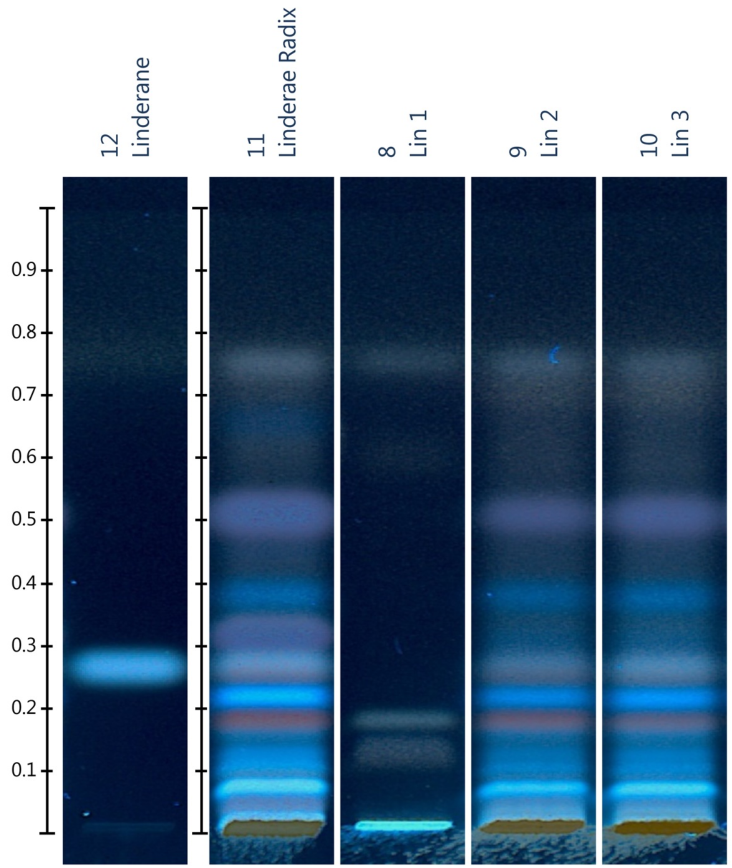

G. Linderae Radix

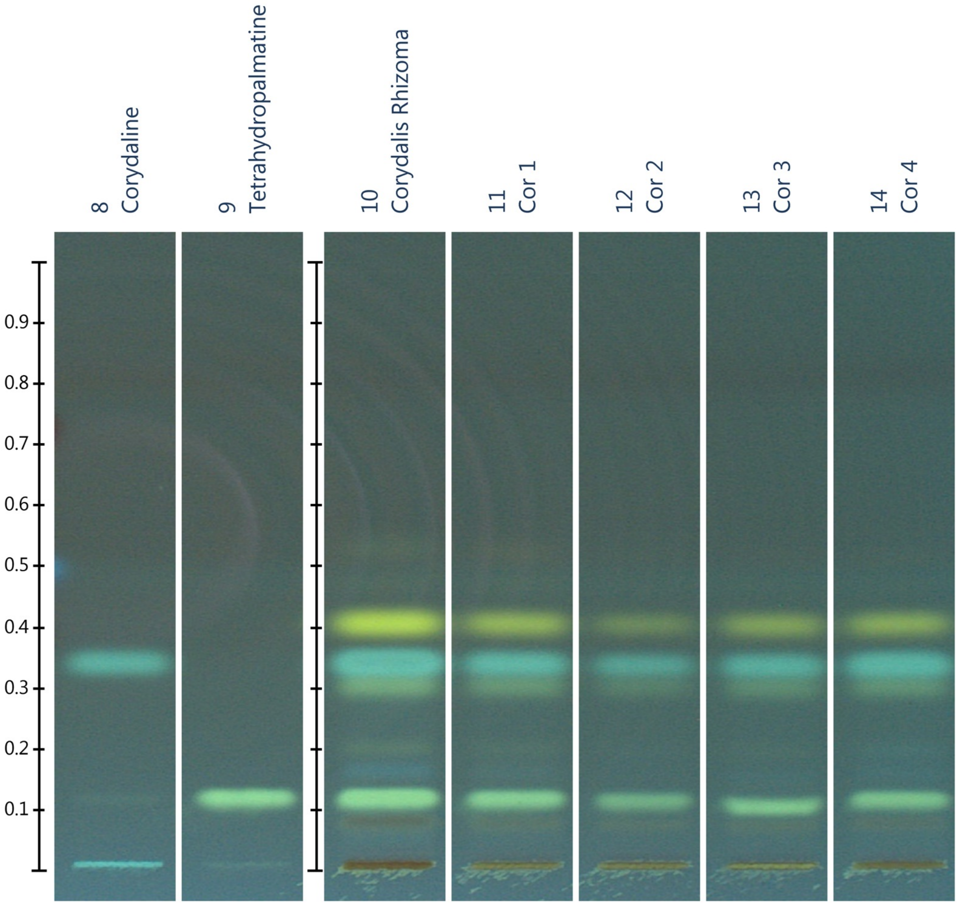

H. Corydalis Rhizoma

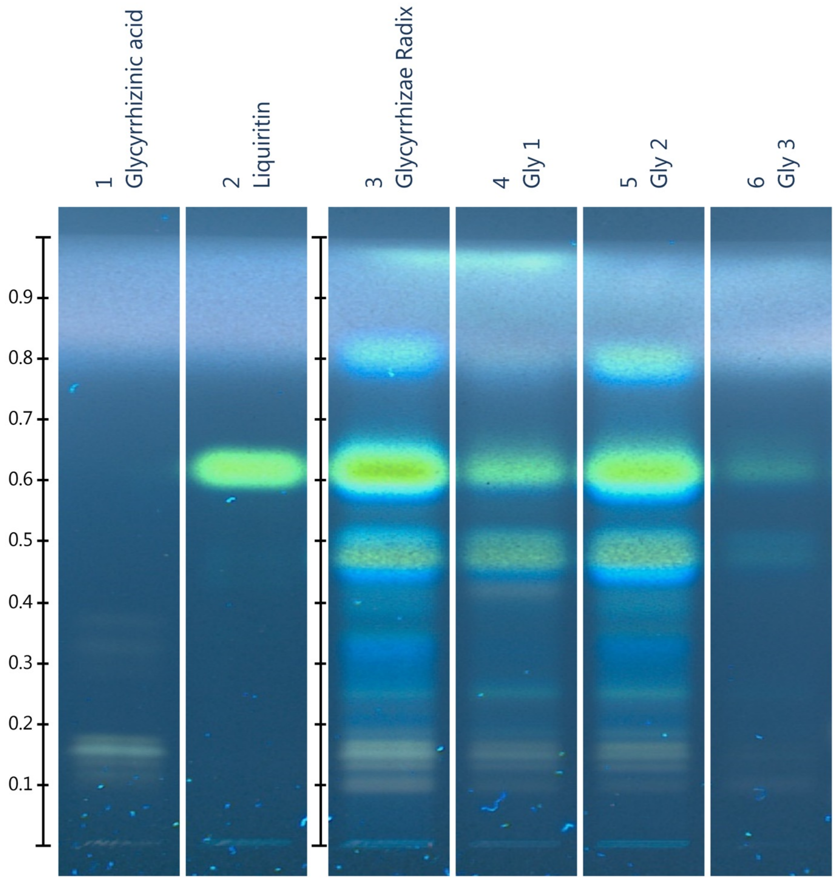

I. Glycyrrhizae Radix

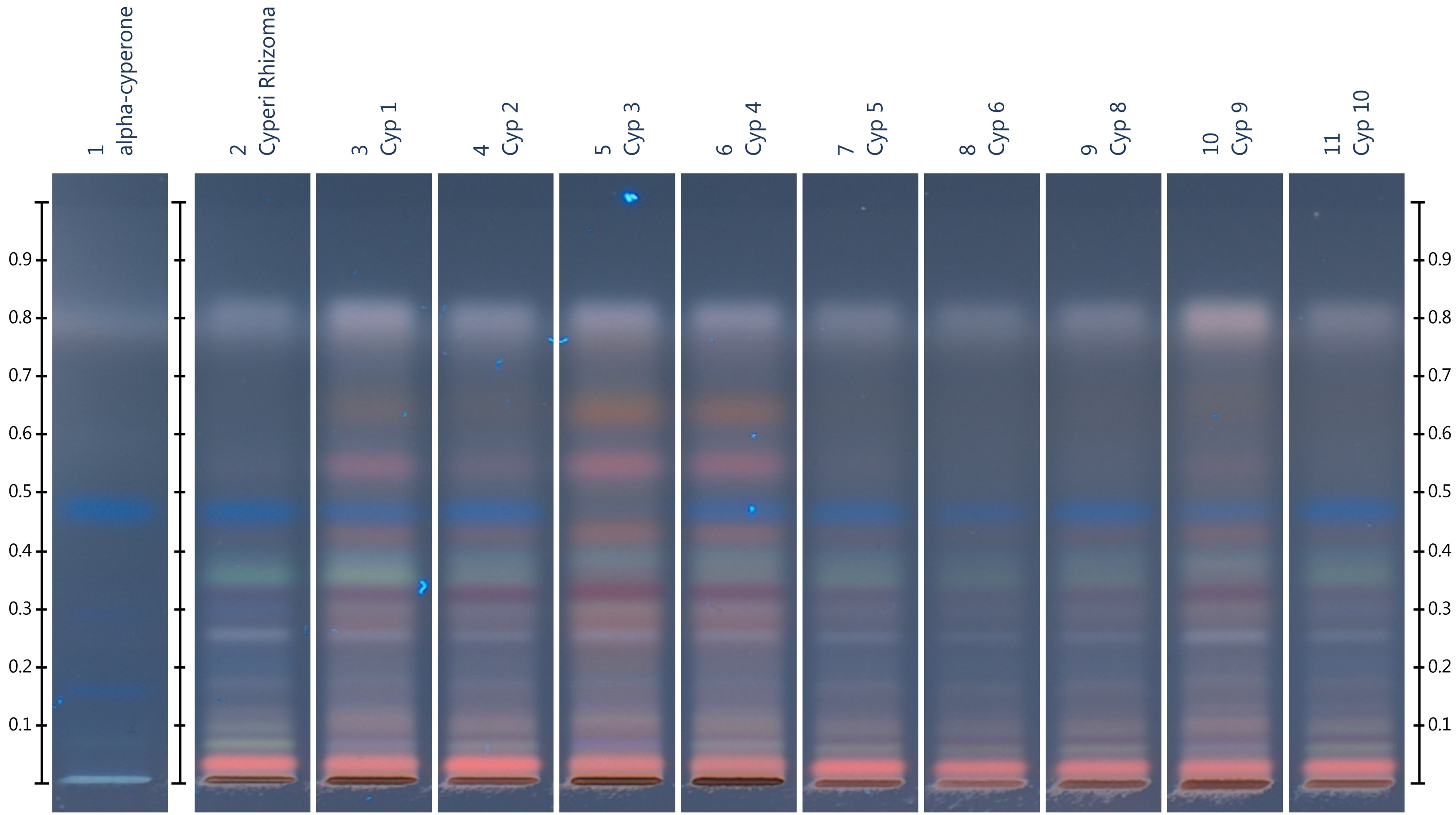

J. Cyperi Rhizoma (Cyp 8-10 is Cyp 7-9)

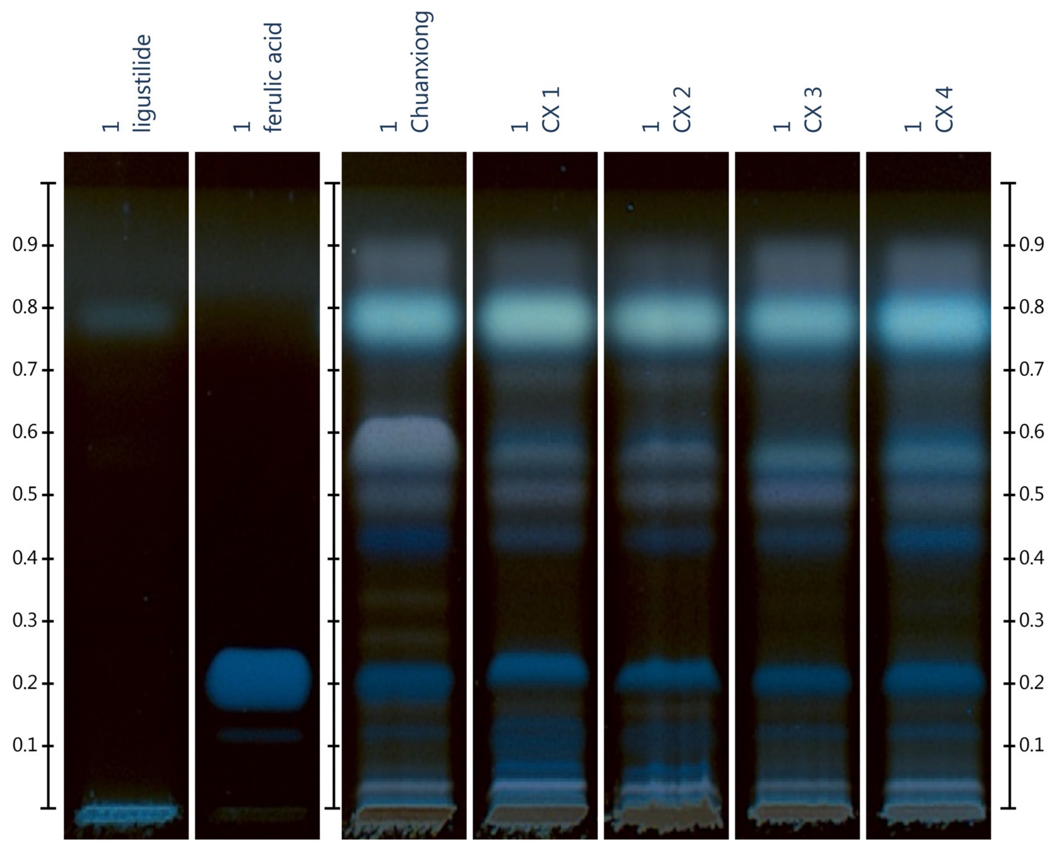

K. Chuanxiong Rhizoma

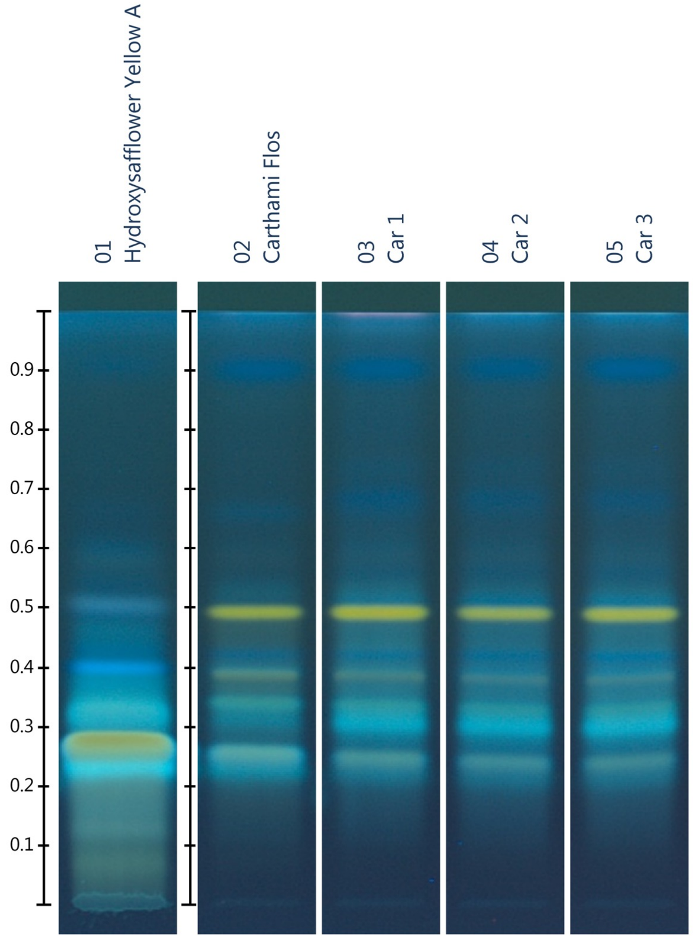

L. Carthami Flos

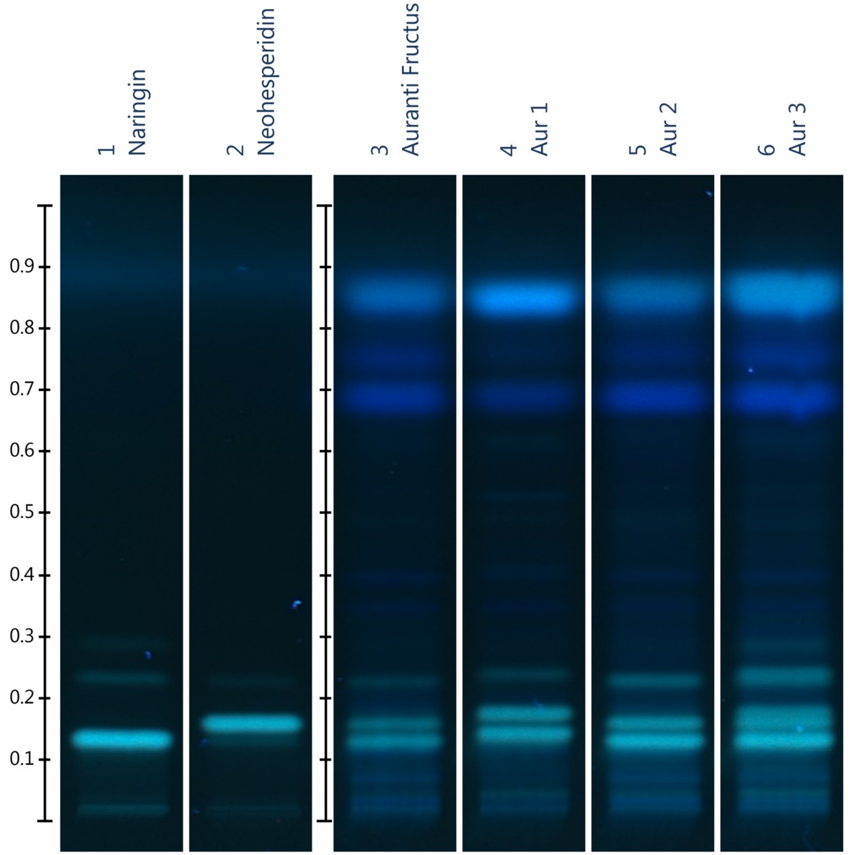

M. Aurantii Fructus
