# Supplementary material for: Three-tiered authentication of herbal traditional Chinese medicine ingredients used in women’s health provides progressive qualitative and quantitative insight
Source: Front Pharmacol. 2024 Feb 5;15:1353434. doi: 10.3389/fphar.2024.1353434 (PMC10875096; doi:10.3389/fphar.2024.1353434)
Supplement: Supplementary file 4 [file DataSheet1.DOCX]

**Supplementary Data S1. HPTLC methods per ingredient (MA- MM)**

HPTLC methods

Samples and marker compounds were applied on HPTLC plates (200.0 x 100.0 mm silica gel 60- F- 254, Merck) as 8.0 mm wide bands using CAMAG Linomat II (CAMAG AG, Mittens, Switzerland). The bands were applied at a distance of 8.0 mm from the lower edge of the plate and 20 mm from the left edge. The development distance is indicated as in each method and is calculated from the lower edge of the plate using CAMAG Automatic Developing Chamber (ADC 2). For derivatization CAMAG derivatizer was used when the derivatizing reagent complied with those optimized by CAMAG, otherwise manual spraying was employed. Each plate was visualized under RT white light and 254 nm and 366nm prior to commencement of the analytical procedure.

MA – Cinnamomi Ramulus

Official name: Cinnamomi Ramulus
Chinese Phonetic Name: Guizhi

Source botanical reference: Cinnamomi Ramulus is the dried young branch of *Cinnamomum cassia* Presl (Lauraceae). The young branch is collected in spring and summer, leaves removed, then dried under the sun; or dried after sliced to obtain Cinnamomi Ramulus.

MA - Sample preparation and plate spotting:

Cinnamaldehyde and cinnamic acid were separately dissolved in ethanol (95%) to obtain 1mg/mL solutions. They were respectively spotted in the quantities of 6 μL and 0.6 μL.

Cinnamomi Ramulus botanical reference: 0.5 mg were dissolved in 5 mL ethanol, and sonicated for 45 min, filtered. Finally, 6 μL were spotted.

F1-20 were prepared according to section 2.1 and 15 μL were spotted.

MA – Development:

The plate was developed over an 8 cm path using a petroleum ether, ethyl acetate and formic acid (8:2:0.2) as the mobile phase. The plate was finally visualized at 254 nm.

MB – Poriae Cocos (European Pharmacopoeia 2012)

Official name: Poriae Cocos

Chinese Phonetic Name: Fuling

Source botanical reference: Dried sclerotium without skin of *Wolfiporia extensa* (Peck) Ginns (syn. *Poria cocos* (Schw.) Wolf; *Wolfiporia* (F.A. Wolf) Ryvarden & Gilb).

MB - Sample preparation and plate spotting:

10 mg of 4-aminobenzoic acid, 10 mg of coumarin and 10 mg of thymol were dissolved in 10 mL of methanol and 5 μL of the mixture was spotted on the plate.

Poria Cocos botanical reference: 1 mg were dissolved in 2 ml ethyl acetate and 3 ml methanol, sonicated for 10 min, and centrifuged at 6000 rpm, 5 μL were spotted.

F1-20 were prepared according to section 2.1 and 15 μL were spotted.

MB – Development:

The plate was developed over a 7 cm path using a cyclohexane, 2-propanol and acetic acid (8:1:1) as the mobile phase. The plate was finally visualised at 254 nm.

MC – Paeoniae Radix Rubra and Moutan Cortex

- Official Name: Radix Paeoniae Rubra

Chinese Phonetic Name: Chishao

Source botanical reference: Dried root of *Paeonia lactiflora* Pall. or *Paeonia veitchii* Lynch (Ranunculaceae). The root is collected in spring or autumn; after removal of the rhizomes, rootlets and soil, the root is dried under the sun to obtain Radix Paeoniae Rubra.

- Official Name: Cortex Moutan

Chinese Phonetic Name: Mudanpi

Source botanical reference: Dried root bark of *Paeonia suffruticosa* Andr. (Fam. Ranunculaceae). The root is collected in autumn, the rootlets removed, the bark stripped off, then dried in the sun to obtain Cortex Moutan.

MC - Sample preparation and plate spotting:

Paeoniflorin was dissolved in methanol to obtain 2 mg/mL concentration, while paeonol was dissolved to obtain 2 mg/mL solution in ethyl acetate. 3 μL of the mixture was spotted on the plate.

Paeonia Radix Rubra and Moutan Cortex botanical references: 0.5 g were dissolved in 10 mL methanol, sonicated 30 min, filtered, evaporated to dryness, dissolved in 1 mL methanol. 2 μL were spotted. F1-20 and G1-7 were prepared according to section 2.1 and 15 μL were spotted.

MC – Development:

The plate was developed over a 7 cm path using a mix of chloroform, ethyl acetate, methanol, formic acid (40:5:10:0.2) as the mobile phase. The plate was visualised at 254 nm and then derivatized using a freshly prepared reagent mixture consisting of 10 mL of 2% hydroxybenzaldehyde in methanol and 1 mL of a 50% v/v sulfuric acid in methanol solution. The plate was heated at 125°C until the spots became visible. The plate was then visualised under RT white light.

MD - Persicae Semen

Official Name: Persicae Semen
Chinese Phonetic Name: Taoren

Source botanical reference: Persicae Semen is the dried ripe seed of *Prunus persica* (L.) Batsch or *Prunus davidiana* (Carr.) Franch. (Rosaceae). The ripe fruit is collected, sarcocarp and endocarp are removed, the seed gathered and then dried under the sun to obtain Persicae Semen.

MD - Sample preparation and plate spotting:

Amygdalin (Ref1) was dissolved in methanol to obtain mg/mL. 5 μL was spotted on the plate.

Persicae semen botanical reference: 25 mL petroleum ether were added to 1 g of sample and sonicated for 1 h. The mixture was filtered with a Buchner Funnel and the filtrate discarded. The residue was sonicated in 10 mL of methanol for 1 hour. Following filtration, the filtrate was made up to 10 mL in a volumetric flask using methanol and 2 μL were spotted. F1-20 and G1-7 were prepared according to section 2.1 and 15 μL were spotted.

MD – Development:

The plate was developed over an 8 cm path using ethyl acetate, methanol and water (40:15:6) as the mobile phase. The plate was visualised at 254 nm. Derivatization was performed by spraying a reagent mixture obtained by pouring slowly 25 mL of sulfuric acid (20% v/v) into 25 mL ice-cold glacial acetic acid. 2.5 mL of p-anisaldehyde were added followed by further 50 mL of sulfuric acid (20% v/v). The plate was then heated at 105°C until the spots became visible. The plate was then visualised under RT white light.

ME – Angelica Sinensis Radix Lipid Fraction

Official Name: Radix Angelicae Sinensis
Chinese Phonetic Name: Danggui

Source botanical reference: Radix Angelicae Sinensis is the dried root of *Angelica sinensis* (Oliv.) Diels (Fam. Apiaceae) (Umbelliferae). The root is usually collected in late autumn after 2 years of plantation, The stems, leaf sheaths, rootlets and soil removed, slightly dried and tied up in small bundles, after placed on a shelf and smoke-dried to obtain Radix Angelicae Sinensis.

ME - Sample preparation and plate spotting:

Z-ligustilide was dissolved in methanol to obtain 1 mg/mL. 4 μL was spotted. Angelica sinensis botanical reference: 10 mL of diethyl ether are added to 1.0 g of sample, the sealed tube was then shaken on an orbital shaker for 2 h. Finally, the sample was centrifuged at 6000 rpm for 10 min. The supernatant was transferred and allowed to evaporate in the air. The residue was taken up in 1 mL ethyl acetate - spot 4 μL. G1-7 were prepared according to section 2.1. 15 μL were spotted.

ME – Development:

The plate was developed over an 8 cm path using hexane and ethyl acetate (5:1) as the mobile phase. The plate was visualised at 254 nm. Derivatization was performed by spraying a 10% solution of sulfuric acid in ethanol. The plate was heated at 110°C for 10 min and then visualised under RT white light and 366 nm.

MF – Angelica Sinensis Radix Water Fraction

MF - Sample preparation and plate spotting:

Ferulic acid was dissolved in methanol to obtain 0.5 mg/mL. 4 μL. Angelica sinensis botanical reference: 20 mL of methanol were added to 2.0 g of sample and the mixture sonicated for 30 min, then centrifuged for 10 min at 6000 rpm. The supernatant was evaporated to dryness with a rotary evaporator. The residue was taken up in 20 mL water and extracted twice with 20 mL of hexane, discarding the hexane layers. The aqueous layer’s pH was adjusted to 2 using 2M HCl, followed by extraction with 10 mL diethyl ether, for three times. The organic layers were combined and evaporated to dryness with a rotary evaporator. The residue was dissolved in 0.5 mL ethyl acetate. 4 μL were spotted. G1-7 were prepared according to section 2.1 and 15 μL were spotted.

MF – Development: The plate was developed over an 8.5 cm path using chloroform, ethyl acetate and formic acid (10:5:0.5) as the mobile phase. The plate was visualised at 254 nm. Derivatization was performed by spraying a 10% solution of sulfuric acid in ethanol. The plate was heated at 110°C for 10 min and then visualised under RT white light and 366 nm.

MG – Linderae Radix

Official Name: Linderae Radix
Chinese Phonetic Name: Wuyao

Source botanical reference: Linderae Radix is the dried root tuber of *Lindera aggregata* (Sims) Kosterm. (Lauraceae). The root tuber is collected all year round, rootlets removed, washed clean, then dried under the sun. Alternatively, the root tuber is sliced while fresh, and dried under the sun to obtain sliced form of Linderae Radix.

MG - Sample preparation and plate spotting:

Linderane was dissolved in methanol to obtain 0.75 mg/mL. 6 μL were spotted. Linderae Radix botanical reference: 15 mL of methanol were added to 0.5 g of sample and the mixture sonicated for 40 min. The suspension was filtered using Millex PES 0.22 μm syringe filters and evaporated to dryness with a rotary evaporator. The residue was dissolved in 0.5 mL of methanol. 6 μL were spotted. G1-7 were prepared according to section 2.1 and 15 μL were spotted.

MG – Development:

The plate was developed over an 8 cm path using n-hexane and ethyl acetate (5:1) as the mobile phase. The plate was visualised at 254 nm. Derivatization was performed using a vanillin reagent prepared by adding 2 mL of sulfuric acid to 100 mL of a 10 g/L solution of vanillin in ethanol (96%). The plate was heated at 105°C until the spots became visible and then visualised under RT white light.

MH – Corydalis Rhizoma

Official Name: Corydalis Rhizoma
Chinese Phonetic Name: Yanhusuo

Source botanical reference: Corydalis Rhizoma is the dried tuber of *Corydalis yanhusuo* W. T. Wang (Papaveraceae). The tuber is collected in early summer when the plant withers, the fibrous root is removed, washed clean, boiled in water until no dry core is visible, then dried under the sun to obtain Corydalis Rhizoma.

MH - Sample preparation and plate spotting:

Corydaline (Ref1) was dissolved in 100% ethanol to obtain 0.1 mg/mL. 0.5 μL Tetrahydropalmatine (Ref 2) was dissolved in 100% methanol to obtain 0.1 mg/mL. 0.5 μL were spotted. Corydalis Rhizoma botanical reference: 10 mL of 70% ethanol were added to 1 g of sample and the mixture sonicated for 10 min. The suspension was finally filtered using Millex PES 0.22 μm syringe filters. 1 μL were spotted. G1-7 were prepared according to section 2.1 and 15 μL were spotted.

MH – Development:

The plate was developed over an cm path using petroleum ether, ethyl acetate and isopropanol (8:2:1) as the mobile phase. The plate was visualized at 254 nm. Derivatization was performed by iodine vapor fumigation for 3 min, or until the spots became visible. The plate was visualized under RT white light and 366 nm.

MI – Glycyrrhizae Radix

Official Name: Radix et Rhizoma Glycyrrhizae
Chinese Phonetic Name: Gancao

Source botanical reference: Radix et Rhizoma Glycyrrhizae is the dried root and rhizome of *Glycyrrhiza uralensis* Fisch. or *Glycyrrhiza inflata* Bat. (Leguminosae). The root together with the rhizome is collected in the spring and autumn. After removal of the rootlets, the root and rhizome are dried under the sun to obtain Radix et Rhizoma Glycyrrhizae.

MI - Sample preparation and plate spotting:

Glycyrrhizic acid and liquiritin were separately dissolved in methanol to obtain 2 mg/mL. 1 μL was spotted. Glycyrrhizae radix botanical reference: 40 mL of diethyl ether were added to 1 g of sample and the mixture refluxed for 1 hour. After cooling to room temperature, the mixture was filtered with a buchner system and the filtrate discarded. The residue was then transferred to a new flask and refluxed for 1 hour in 30 mL of methanol. After cooling to room temperature, the mixture was filtered once again. The filtrate was evaporated to dryness using a rotary evaporator. The residue was taken up in 40 mL of water and extracted three times with 20 mL of 1-butanol each time. The 1-butanol layers were combined and washed three times with 20 mL of water, each time. Finally, the 1-butanol extract was evaporated to dryness with a rotary evaporator and the residue taken up in 5 mL of methanol - spot 2 μL. G1-7 were prepared according to section 2.1 and 15 μL were spotted.

MI – Development:

The plate was developed over an 8 cm path using a mobile phase prepared as follows: 1-butanol, glacial acetic acid and water (7:1:12) were mixed in a separating funnel and left to separate over a period of 30 min. The upper layer was then used as the mobile phase. The plate was visualised at 254 nm. Derivatization was obtained by spraying with a 10% solution of sulfuric acid in ethanol. The plate was heated to 110°C until the spots became visible. The plate was visualised under RT white light.

MJ – Cyperi Rhizoma

Official Name: Cyperi Rhizoma
Chinese Phonetic Name: Xiangfu

Source botanical reference: Cyperi Rhizoma is the dried rhizome of *Cyperus rotundus* L. (Cyperaceae). The rhizome is collected in autumn and fibrous roots are burnt off, dried under the sun; or boiled briefly or steamed thoroughly, then dried under the sun to obtain Cyperi Rhizoma.

MJ - Sample preparation and plate spotting:

α-cyperone was dissolved in methanol to obtain 1 mg/mL. 3 μL were spotted. Cyperi rhizoma botanical reference: 1.5 g were suspended in 10 mL of methanol, sonicated for 30 min and filtered with 0.22 μm Millex PES syringe filters and 6 μL were spotted on the plate. G1-7 were prepared according to section 2.1 and 15 μL were spotted.

MJ – Development:

The plate was developed over a 7 cm path using a mobile phase prepared as follows: n-hexane, ethyl acetate and glacial acetic acid (9:1:0.1). The plate was visualised at 254 nm. Derivatization was obtained by spraying with a p-anisaldehyde-sulfuric acid reagent which was prepared by mixing 0.5 mL of p-anisaldehyde with 10 mL glacial acetic acid, 85 mL of methanol and 5 mL of sulfuric acid. The plate was heated to 100 °C until the spots became visible. The plate was visualised under RT white light.

MK – Chuangxiong Rhizoma

Official Name: Rhizoma Chuanxiong

Chinese Phonetic Name: Chuanxiong

Source botanical reference: Rhizoma Chuanxiong is the dried rhizome of *Ligusticum chuanxiong* Hort. (Apiaceae/Umbelliferae). The rhizome of cultivated plants is harvested in the summer in the second year of cultivation, when bulging nodes on the stem become prominent and slight purplish in colour. The whole plant is excavated, the rhizome and soil removed, then dried in a shaded area or by baking; once dried, the rootlets are removed to obtain Rhizoma Chuanxiong.

MK - Sample preparation and plate spotting:

Z-ligustilide and ferulic acid were separately dissolved in methanol to obtain 1 mg/mL. 1 μL was spotted. Chuang Xiong botanical reference: 10 mL of methanol were added to 1.0 g of sample and the mixture sonicated for 30 min, then centrifuged for 20 min at 6000 rpm. The supernatant was evaporated to dryness with a rotary evaporator. The residue was taken up in 2 mL of methanol. 1 μL was spotted. G1-7 were prepared according to section 2.1 and 15 μL were spotted.

MK – Development:

The plate was developed over a 5 cm path using dichloromethane and diethyl ether (2:1) as the mobile phase. The plate was visualised at 254 nm. Derivatization was performed by spraying a 10% solution of sulfuric acid in ethanol. The plate was heated at 70°until the spots became visible and then visualised under RT white light and 366 nm.

ML – Carthami Flos

Official Name: Carthami Flos
Chinese Phonetic Name: Honghua

Source botanical reference: Carthami Flos is the dried flower of *Carthamus tinctorius* L. (Asteraceae). The flower is collected in summer when it turns from yellow to red, then dried in a shaded area or under the sun to obtain Carthami Flos.

ML - Sample preparation and plate spotting:

Hydroxysafflower yellow A was freshly dissolved in methanol to obtain 1 mg/mL. 2 μL were spotted. Carthami flos botanical reference: 0.1 g were suspended in 1 mL of 80% acetone, sonicated for 15 min, and filtered with 0.22 μm Gilson PTFE syringe filters. 300 μL of filtrate were transferred with 300 μL of acetone in a tube and centrifuged for 1 min at 13500 rpm. 2 μL of the supernatant were spotted on the plate. G1-7 were prepared according to section 2.1 and 15 μL were spotted.

ML – Development:

The plate was developed over a 7 cm path using a mobile phase prepared as follows: ethyl acetate water, formic acid and methanol (7:3:2:0.4). The plate was visualised under white light and at 254 nm.

MM – Aurantii Fructus

Official Name: Aurantii Fructus
Chinese Phonetic Name: Zhiqiao

Source botanical reference: Aurantii Fructus is the dried immature fruit of *Citrus aurantium* L. (Rutaceae) and its cultivated varieties. The immature fruit is collected in July while the fruit is green, cut into two parts, then dried under the sun or at ambient temperature to obtain Aurantii Fructus.

MM - Sample preparation and plate spotting:

Naringin was dissolved in ethanol to obtain 0.5 mg/mL. 10 μL was spotted.

Neohesperidin was dissolved in methanol to obtain 0.5 mg/mL. 10 μL Auranti fructus botanical reference: 0.2 g were suspended in 100 mL of ethanol, sonicated for 30 min, filtered with 0.22 μm Millex PES syringe filters and evaporated. The residue was taken up in 5 mL of ethanol and 10 μL of the supernatant were spotted on the plate. G1-7 were prepared according to section 2.1 and 15 μL were spotted.

MM – Development:

The plate was developed over a 7 cm path using a mobile phase prepared as follows: chloroform, methanol and water (13:6:2). The plate was derivatized with a 2% (m/v) aluminium chloride in ethanol and visualised at 366 nm.
